# Efficacy of Antiviral Therapy in Chronic Hepatitis B Patients With Normal Alanine Aminotransferase: A Systematic Review and Meta-Analysis

**DOI:** 10.1155/cjgh/7689981

**Published:** 2025-03-08

**Authors:** Yuting Diao, Yueying Zeng, Zhihao Huang, Chunfang You

**Affiliations:** Department of Infectious Diseases, Zigong First People's Hospital, 42, Shangyihao Branch Road 1, Zigong 643000, Sichuan, China

**Keywords:** alanine aminotransferase, antiviral efficacy, hepatitis B virus, meta-analysis, systematic review

## Abstract

**Background and objectives:** The efficacy of antiviral therapy in chronic hepatitis B (CHB) patients with normal alanine aminotransferase (ALT) is controversial. This study aimed to systematically review and analyze antiviral efficacy in ALT-normal CHB patients.

**Methods:** PubMed, Embase, Web of Science, and the Cochrane Library databases from inception to 17 May 2024 were searched for retrieving relevant studies with antiviral efficacy of ALT-normal CHB patients.

**Results:** Of 4992 records screened, 10 studies met the criteria for inclusion and had a low risk of bias. The pooled proportions of undetectable HBV DNA, HBeAg loss, HBeAg seroconversion, HBsAg loss, and HBsAg seroconversion in ALT-normal CHB patients with antiviral therapy were 87%, 35%, 19%, 16%, and 10%, respectively. Subgroup analysis suggested that the virological and serological responses were better in patients receiving IFN-based therapy or with a longer follow-up time. Compared with no treatment, antiviral therapy was associated with significant higher rates of undetectable HBV DNA (RR: 65.62, 95% CI: 16.65–258.57, and *p* < 0.01), HBeAg loss (RR: 14.97, 95% CI: 3.31–67.65, and *p* < 0.01), HBsAg loss (RR: 14.22, 95% CI: 4.10–49.29, and *p* < 0.01), and HBsAg seroconversion (RR: 24.65, 95% CI: 3.06–198.60, and *p* < 0.01). The normal ALT group and elevated ALT group had comparable antiviral efficacy including proportions of undetectable HBV DNA, HBeAg loss, and HBeAg seroconversion (*p* > 0.05).

**Conclusions:** CHB patients with normal ALT could benefit from antiviral therapy, and the virological and serological responses were comparable to that of ALT-elevated ones.

## 1. Introduction

Chronic hepatitis B (CHB) remains a burdensome public health problem. Although no complete cure strategy exists for HBV infection, antiviral therapy can effectively inhibit HBV replication and even achieve functional cure [1]. In 2016, the World Health Organization (WHO) proposed the goal of eliminating viral hepatitis as a major public health hazard by 2030. The 2030 targets include diagnosis of 90% of people infected with HBV and antiviral treatment of 80% of those diagnosed and eligible for treatment [2]. A study from the US evaluating treatment eligibility among 84,916 CHB patients reported that only 6.7%, 6.2%, and 5.8% of the participants met the American Association for the Study of Liver Disease (AASLD), European Association for Study of the Liver (EASL), and Asian Pacific Association for Study of the Liver (APASL) criteria, respectively [3]. This spelled that it was impossible to achieve the WHO's goal of achieving an 80% CHB treatment rate by following the treatment standards of the three guidelines.

Currently, many authoritative guidelines have gradually relaxed the indications for antiviral therapy. For example, 8%–15% of the patients met the treatment criteria of the 2015 WHO guidelines [4], while more than 50% met that of the 2024 WHO guidelines [5]. But a considerable number of CHB patients still have not received antiviral therapy because they do not meet the established standards. A multicenter retrospective cohort study followed up 3624 untreated patients with chronic HBV infection, of which 161 had hepatocellular carcinoma (HCC). The proportions of patients who developed HCC outside treatment recommendation according to APASL, AASLD, and EASL criteria were 64.0%, 46.0%, and 33.5%, respectively [6]. Most of the guidelines set the alanine aminotransferase (ALT) treatment threshold as > 2 × upper limit of normal (ULN) [7–9], and the latest Chinese guidelines set it as > 1 × ULN [10], which is the closest to “treat all.” A total of 94% of the patients met the treatment criteria of the 2022 Chinese guidelines [10]. Furthermore, the existing guidelines lack focus on patient-related issues such as extrahepatic diseases, the patient's reported outcomes, stigmatization, infectivity, quality of life, and patient's willingness. This highlights the urgency of updating the guidelines. Therefore, we conducted a systematic review and meta-analysis to identify the antiviral efficacy in ALT-normal CHB patients and compare the antiviral efficacy in CHB patients with normal and elevated ALT.

## 2. Methods

### 2.1. Search Strategy

This systematic review and meta-analysis were conducted and reported according to the Preferred Reporting Items for Systematic Reviews and Meta-Analyses (PRISMA) guidelines, which was listed in Supporting Table S1. We systematically searched PubMed, Embase, Web of Science, and Cochrane Library from the database inception to 17 May 2024. The major search keywords included (‘hepatitis B' or ‘chronic hepatitis B' or ‘hepatitis B virus'), (‘alanine aminotransferase' or ‘alanine transaminase') and (‘treatment outcome' or ‘efficacy'), and relevant medical terms were retrieved from the MeSH. The detailed search strategy described in the Supporting Information (P1). To assess the quality of the records and avoid bias, we excluded literature including editorials, case reports, reviews, letters, and animal experiments. To identify eligible studies, records were initially screened by title and abstract and then full text. The forward and backward citation tracking was also performed for included records.

### 2.2. Inclusion and Exclusion Criteria

The meta-analysis included studies that (a) contained a group of adult patients both with chronic hepatitis B and with a normal ALT level; (b) divided ALT-normal CHB patients into antiviral treatment group (treated with nucleos (t) ide analogs (NAs) and/or interferon (IFN)) and nonantiviral treatment group (no treatment or placebo), or divided CHB patients with antiviral treatment (treated with NAs and/or IFN) into ALT-normal group and ALT-elevated group; and (c) analyzed the rates of undetectable HBV DNA, HBeAg loss, HBeAg seroconversion, HBsAg loss, or HBsAg seroconversion. Exclusion criteria were as follows: (a) editorials, case reports, reviews, letters, basic experiments or animal studies, or practice guidelines; (b) studies conducted in patients with severe comorbidities such as hepatitis *C* virus, hepatitis *D* virus, human immunodeficiency virus, or use of immunosuppressive therapies; (c) studies that did not exclude prior hepatocellular carcinoma or other cancers; and (d) non-English articles.

### 2.3. Data Extraction and Quality Assessment

Two authors (any two of YTD, YYZ, or ZHH) independently screened potential records and extracted data from eligible ones. Differences were resolved by discussing and reaching a consensus, and a third author joined in if necessary. Inclusion data: publication year, first author's name, area, sample size, participants' age and gender, inclusion and exclusion criteria, baseline ALT level, ULN of ALT, baseline HBeAg-positive rate, baseline HBV DNA level, treatment details, follow-up time, and outcomes (the rates of undetectable HBV DNA, HBeAg loss, HBeAg seroconversion, HBsAg loss, or HBsAg seroconversion).

We assessed the quality of included records using an assessment scale based on the Newcastle–Ottawa scale which comprised of three domains: selection of participants (point from 0 to 4), comparability of study groups (point from 0 to 4), and the ascertainment of outcomes of interest (point from 0 to 3) [11]. Studies with a score of 7–9 were high quality (low risk of bias), those with a score of 4–6 were fair quality (moderate risk of bias), and those with a score of 1–3 were low quality (high risk of bias) [11]. Two authors (any two of YTD, YYZ, or ZHH) independently assessed the risk of bias. In case of disagreement, a third author was invited for consultation if necessary.

### 2.4. Statistical Analysis

Meta-analysis was performed to calculate pooled estimates for the following five outcomes, which were reported as dichotomous variables: undetectable HBV DNA, HBeAg loss, HBeAg seroconversion, HBsAg loss, or HBsAg seroconversion. When two groups were compared, the pooled outcome was calculated as risk ratio (RR) with 95% confidence interval (CI) for abovementioned indicators using the Mantel–Haenszel method. We estimated heterogeneity between studies using Cochran's *Q* statistic (*p* < 0.05 indicated heterogeneity) and the *I*^2^ statistic. A fixed-effects model was used to summarize the data when the heterogeneity was nonsignificant (*I*^2^ ≤ 50%), and a random-effects model was used when the heterogeneity was significant (*I*^2^ > 50%) [12]. Subgroup analysis according to treatment regimen or follow-up time was conducted. Funnel plot and trim-and-fill method were used to assess and correct publication bias when five or more studies were included. Sensitivity analysis was performed to test each individual study's contribution to the pooled results using the “leave-one-out” method. All statistical analyses were conducted using the meta package and metafor package in *R* statistical software (Version 4.1.3).

## 3. Results

### 3.1. Study Selection

**Figure 1:**
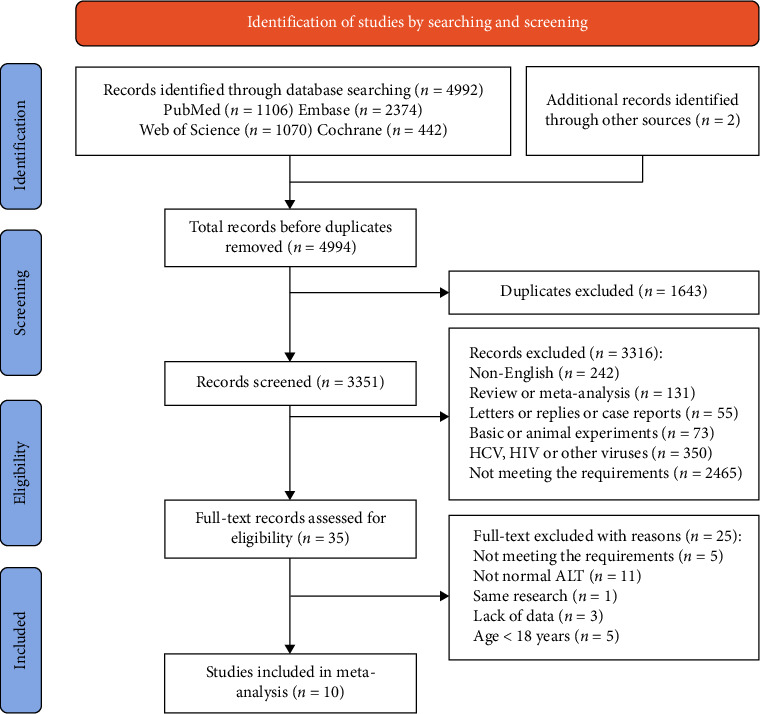
Flowchart of systematic literature search and screening for studies. ALT, alanine aminotransferase; HCV, hepatitis C virus; HIV, human immunodeficiency virus.

A total of 4992 records were retrieved using our search strategy, and 2 additional records were identified through retrospective searching. Following the exclusion of 1643 duplicates, we screened the titles and abstracts of 3351 studies and identified 35 potentially relevant studies. These 35 studies were pulled for full-text review, which eventually yielded 10 eligible studies involving 8 articles [13–20] and 2 abstracts [21, 22] (Figure 1). Five studies compared the virological outcomes of ALT-normal CHB patients with and without antiviral therapy [15–17, 20, 21], four compared the antiviral efficacy of CHB patients with normal and elevated ALT [14, 18, 19, 22], and one did the both [13].

### 3.2. Characteristics of Included Studies

The main characteristics of the included studies were summarized in Table 1. Most of the included studies were conducted in Asia, except for one study [13], which was a multinational cooperative study. One study was published in 2002 [13], and the remaining studies were published between 2013 and 2023. There was a total of 1538 adult participants enrolled from the 10 included studies. The proportions of men were generally higher than that of women, while one article only described postpartum women [16] and one did not show the proportion [21]. The ULN of ALT value varied from 40 U/L to 50 U/L. Five studies enrolled both HBeAg-positive and HBeAg-negative patients with high viral load (HBV DNA > 20,000 IU/mL) in most of them [14, 15, 18, 19, 22], three involved HBeAg-negative patients with low viral load (HBV DNA ≤ 20,000 U/mL) in most of them [17, 20, 21], and two involved HBeAg-positive patients [13, 16]. Of the 10 identified records, four studies involved interferon monotherapy or combination therapy [13, 16, 17, 21], others used NAs for anti-HBV treatment. The follow-up periods for the cohort studies ranged from 24 weeks to more than 60 months.

The quality assessment was shown in Supporting Table S2. Most of the included studies were of good quality with quality assessment scores greater than 7, and only two were of fair quality with scores 5 or 6 [18, 19]. None of these studies got low-quality scores.

### 3.3. Virological Response of ALT-Normal CHB Patients

**Figure 2:**
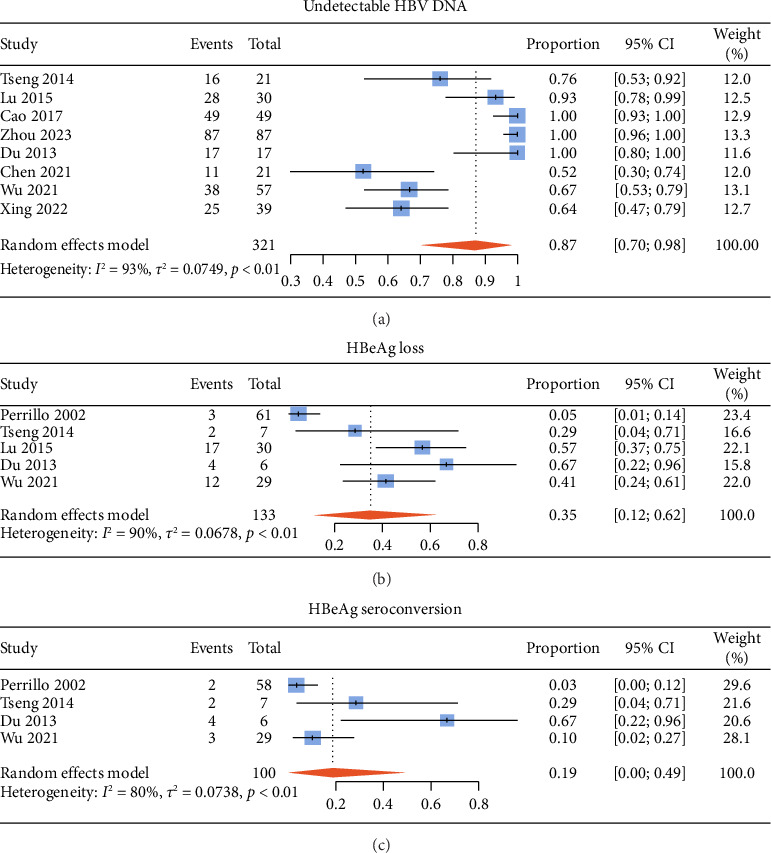
Pooled proportions of undetectable HBV DNA (a), HBeAg loss (b), and HBeAg seroconversion (c) in ALT-normal CHB patients with antiviral therapy. DNA, deoxyribonucleic acid; HBeAg, hepatitis B envelope antigen.

The pooled proportion of undetectable HBV DNA in ALT-normal CHB patients with antiviral therapy was 87% (95% CI: 70%–98%) (Figure 2(a)), that of HBeAg loss was 35% (95% CI: 12%–62%) (Figure 2(b)), and that of HBeAg seroconversion was 19% (95% CI: 0%–49%) (Figure 2(c)). The pooled proportion of HBsAg loss and HBsAg seroconversion was 16% (95% CI: 2%–38%) and 10% (95% CI: 0%–33%), respectively (Supporting Figures S1a and S1b).

We performed subgroup analyses based on treatment regimen or follow-up time (Supporting Figures S3–S7). The proportion of undetectable HBV DNA in patients receiving IFN-based therapy was nonstatistically higher than that in patients with IFN-free regimen (*p* > 0.05). HBeAg seroconversion, HBsAg loss, and HBsAg seroconversion showed the same trend, and the differences were statistically significant (*p* < 0.05), while HBeAg loss showed the opposite trend (*p* < 0.05). The proportion of undetectable HBV DNA in patients with a follow-up time no shorter than 96 weeks was significantly higher than that in patients with a follow-up time shorter than 96 weeks, the same of HBeAg loss and HBeAg seroconversion (*p* < 0.05). HBsAg loss and HBsAg seroconversion also showed the same trend, but the differences were not statistically significant (*p* > 0.05).

### 3.4. Virological Outcomes of Treated Versus Untreated ALT-Normal CHB Patients

**Figure 3:**
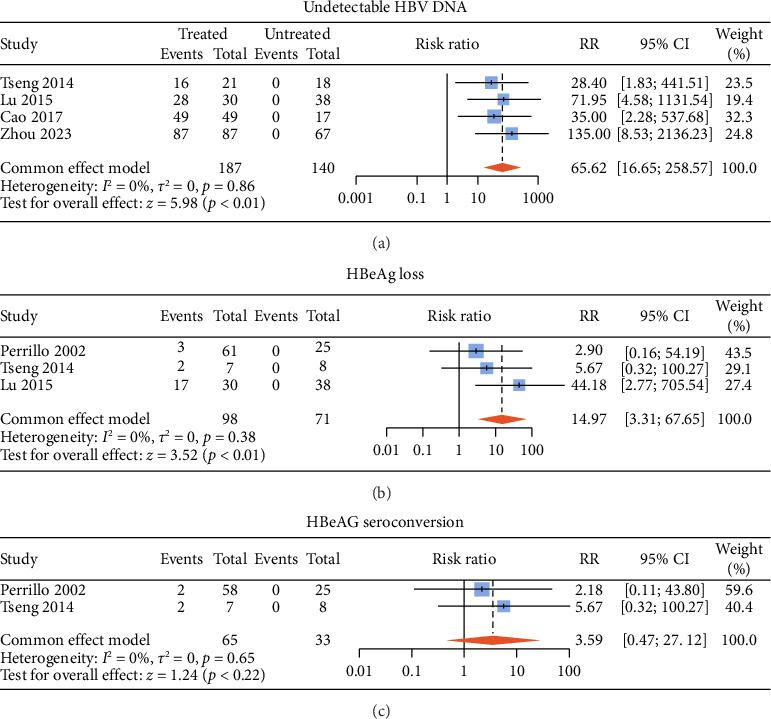
Pooled risk ratios for undetectable HBV DNA (a), HBeAg loss (b), and HBeAg seroconversion (c) between the treated group and untreated group. DNA, deoxyribonucleic acid; HBeAg, hepatitis B envelope antigen; RR, risk ratio.

There were six studies [13, 15–17, 20, 21] comparing the virological outcomes of ALT-normal CHB patients with and without antiviral therapy. Among these studies, four studies [15–17, 20] described undetectable HBV DNA, three [13, 15, 16] described HBeAg loss, and two [13, 15] described HBeAg seroconversion. Compared with no treatment, antiviral therapy was associated with significant difference in the rates of undetectable HBV DNA (RR: 65.62, 95% CI: 16.65–258.57, and *p* < 0.01; *I*^2^ = 0) (Figure 3(a)) and HBeAg loss (RR: 14.97, 95% CI: 3.31–67.65, and *p* < 0.01; *I*^2^ = 0) (Figure 3(b)) without noticeable heterogeneity and so did HBsAg loss (RR: 14.22, 95% CI: 4.10–49.29, and *p* < 0.01; *I*^2^ = 0) (Supporting Figure S2a) and HBsAg seroconversion (RR: 24.65, 95% CI: 3.06–198.60, and *p* < 0.01; *I*^2^ = 0) (Supporting Figure S2b). However, there was no statistical difference in HBeAg seroconversion between the two groups (RR: 3.59, 95% CI: 0.47–27.12, and *p*=0.22; *I*^2^ = 0) (Figure 3(c)).

### 3.5. Antiviral Efficacy of ALT-Normal Versus ALT-Elevated CHB Patients

**Figure 4:**
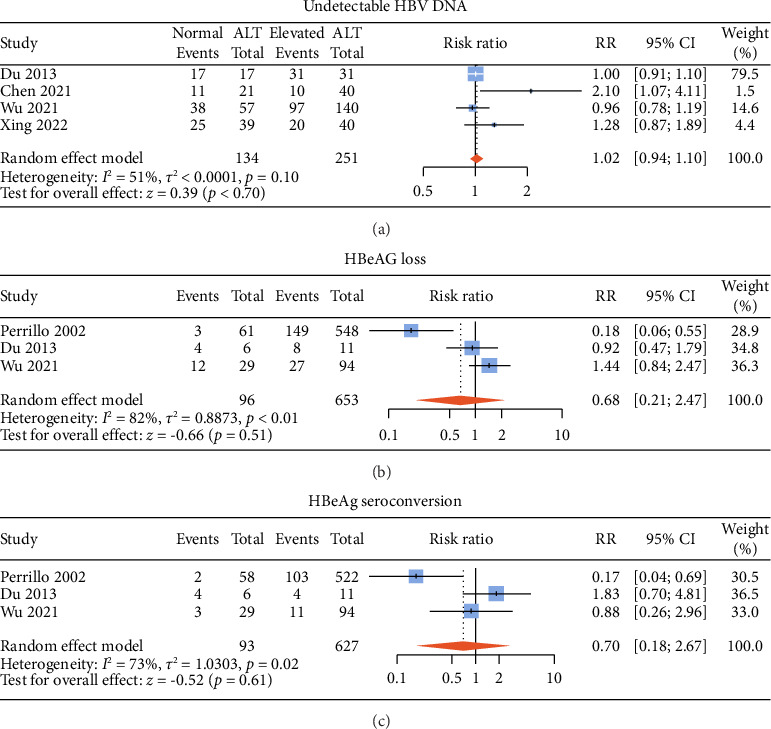
Pooled risk ratios for undetectable HBV DNA (a), HBeAg loss (b), and HBeAg seroconversion (c) between the ALT-normal and ALT-elevated CHB patients with antiviral therapy. ALT, alanine aminotransferase; DNA, deoxyribonucleic acid; HBeAg, hepatitis B envelope antigen; RR, risk ratio.

There were five studies [13, 14, 18, 19, 22] comparing the antiviral efficacy of CHB patients with normal ALT and those with elevated ALT. Among these studies, four studies [14, 18, 19, 22] described undetectable HBV DNA, three [13, 14, 19] described HBeAg loss, and three [13, 14, 19] described HBeAg seroconversion. Normal ALT group and elevated ALT group had comparable proportions of undetectable HBV DNA (RR: 1.02, 95% CI: 0.94–1.10, and *p*=0.70; *I*^2^ = 51%) (Figure 4(a)), HBeAg loss (RR: 0.68, 95% CI: 0.21–2.15, and *p*=0.51; *I*^2^ = 82%) (Figure 4(b)) and HBeAg seroconversion between the two groups (RR: 0.70, 95% CI: 0.18–2.67, and *p*=0.61; *I*^2^ = 73%) (Figure 4(c)) with noticeable heterogeneity. Since only one study described HBsAg loss and HBsAg seroconversion [14], meta-analysis of these two indicators could not be done.

### 3.6. Publication Bias and Sensitivity Analysis

When the number of included studies was no less than 5, funnel plot and trim-and-fill method were carried out (Supporting Figures S8–S10 and Supporting Tables S3–S5). In addition, we further produced sensitivity analysis diagrams by “leave-one-out” (Supporting Figures S11–S13).

### 3.7. Supporting Material

Supporting Table S1 listed the PRISMA statement. The Newcastle–Ottawa Quality Assessment Scale of included studies was shown in Supporting Table S2. The trim-and-fill method was carried out for pooled proportions of undetectable HBV DNA (Supporting Table S3), HBsAg loss (Supporting Table S4), and HBeAg loss (Supporting Table S5).

The pooled proportions of HBsAg loss and HBsAg seroconversion in ALT-normal CHB patients with antiviral therapy were shown in Supporting Figures S1a and S1b. Supporting Figures S3–S7 showed subgroup analysis based on treatment regimen or follow-up time in ALT-normal CHB patients with antiviral therapy. Supporting Figure S2 showed pooled RRs for HBsAg loss and HBsAg seroconversion between the treated group and untreated group. Funnel plots were performed for pooled proportions of undetectable HBV DNA (Supporting Figure S8), HBsAg loss (Supporting Figure S9), and HBeAg loss (Supporting Figure S10). We further produced sensitivity analysis diagrams by “leave-one-out” (Supporting Figures S11–S13).

## 4. Discussion

To the best of our knowledge, this was the first systematic review and meta-analysis focusing on the efficacy of antiviral therapy in CHB patients with normal ALT. In this article, we evaluated the pooled proportions of undetectable HBV DNA, HBeAg loss, HBeAg seroconversion, HBsAg loss, and HBsAg seroconversion in ALT-normal CHB patients with antiviral therapy. Our pooled analysis indicated that antiviral therapy increased the probability of undetectable DNA, HBeAg loss, and HBeAg seroconversion in ALT-normal CHB patients, and these virological responses were comparable with those in ALT-elevated CHB patients with antiviral therapy. We also found that with the duration extension of antiviral treatment or the use of IFN, the efficacy might go better.

Currently, there is no guideline for CHB management including ALT-normal CHB patients without liver fibrosis or cirrhosis into the population recommended for antiviral therapy. However, ALT within the normal range does not mean an individual has no histological lesions. One study found that among CHB patients with normal ALT, more than 30% had abnormal liver histology, including significant inflammation and fibrosis [19]. A recent meta-analysis including 33 studies reported that the estimated prevalences of non-fibrosis, significant fibrosis, advanced fibrosis, and cirrhosis for HBeAg-positive ALT-normal CHB were 31.2%, 16.9%, 5.4%, and 0.0% and for HBeAg-negative ALT-normal CHB were 32.4%, 24.8%, 3.0% and 0.0%, respectively [23]. ALT level was associated with liver related events including HCC in CHB patients even with normal ALT. The REVEAL study has revealed that ALT ≥ 15 IU/L was associated with a higher risk for HCC development in treatment-naïve CHB patients with normal ALT [24]. Antiviral therapy has been shown to reduce the incidence of liver-related events in CHB, including cirrhosis, end-stage liver disease, and HCC, regardless of ALT levels [25–27]. Untreated CHB patients with normal ALT had higher risks of HCC and death/transplantation than treated patients with ALT ≥ 2 × ULN no matter the HBeAg status [25, 26]. In a cohort study of 3665 CHB patients without cirrhosis, the incidence of HCC was significantly reduced in both groups of patients with ALT < 2 × ULN or ≥ 2 × ULN after antiviral therapy, and there was no significant difference in the degree of reduction in HCC incidence between the two groups [27]. However, there were currently conflicting conclusions. Some studies suggested that untreated CHB patients with normal ALT levels had good long-term prognosis and a low risk of developing cirrhosis or HCC [28, 29].

The antiviral efficacy of CHB patients with normal ALT has been confirmed in recent studies. The study of Gao et al. found that NAs monotherapy has a good virological response in ALT-normal CHB patients. In patients with low baseline viral load, the rates of undetectable HBV DNA was higher than 90% at 4 weeks of treatment and even up to 100% at 12 weeks [30]. The study of Wei et al. showed that during the 96 weeks of the study, patients with normal ALT could achieve viral suppression similar to those with mildly elevated ALT, and the liver stiffness values of the two groups were significantly improved after antiviral therapy [31]. A recent meta-analysis including CHB patients with ALT level < 2 × ULN indicated that antiviral treatment could significantly improve the HBsAg loss rate, and IFN played an important role in HBsAg loss with or without HBsAg seroconversion than Nas [32]. Our study yielded similar conclusions in the population of ALT-normal CHB patients. Therefore, it is necessary and feasible to expand the indications for antiviral therapy.

The current standard-of-care medications for CHB include NAs and IFN [33]. NAs can potently inhibit replication of HBV and reduce viral load to undetectable level after 48 weeks of therapy in most patients but rarely reduce HBsAg levels [34]. In contrast, a finite duration of IFN-based treatment has been shown to be superior to NAs monotherapy in achieving higher rates of HBeAg clearance and HBsAg loss [35]. The present study showed the same results. In the subgroup analysis stratified by treatment regimen, IFN-based therapy, compared with IFN-free therapy, could improve the efficacy of HBsAg loss and HBsAg seroconversion. However, this advantage did not appear in HBeAg loss or seroconversion. The possible reasons for the difference were that the limited number of eligible studies and miscellaneous treatment strategies in one study [13]. With the extension of treatment duration, rates of undetectable HBV DNA, HBeAg loss, and HBeAg seroconversion increased. However, due to the limited number of studies, HBeAg loss or seroconversion did not show the same trend. Furthermore, there are many factors that affect the antiviral efficacy of CHB patients, including age, genetic susceptibility, baseline of virological indicators, HBV genotype, ALT level, and fatty liver [36–38]. More studies are needed to further explore the impacts of these factors.

The present systematic review and meta-analysis has several limitations which should be considered during the interpretation of the findings. First, the meta-analysis only yielded few studies, which impeded further subgroup analysis to explore the influences of some confounding factors. In addition, there were not enough participants included in the eligible records, with a total sample size of 1 538, which could lead to unstable estimates. Second, because of the ULN of ALT was inconsistent in some studies or not mentioned in others, and no further grouping based on ALT levels, it was infeasible to further analyze the efficacy of antiviral therapy for CHB patients with high-normal ALT or low-normal ALT. Third, the included studies were also heterogeneous in their study populations, HBV genotypes, HBeAg status, HBV DNA levels, treatment strategies, testing methods of virological indicators, and follow-up duration. There fore, we performed sensitivity analyses to improve the stability, reliability, and accuracy of the analyses.

In conclusion, antiviral therapy enabled CHB patients with normal ALT to achieve good virological and serological responses, which was not inferior to that in patients with elevated ALT. Furthermore, prolonged duration of antiviral therapy and IFN-containing regimens were conducive to inhibition of HBV. Our results provide preliminary evidence for the “treat all” strategy of CHB. Future studies with larger sample size, longer follow-up, and adequate adjustment for potential confounders are still required.

## Figures and Tables

**Table 1:** Characteristics of included studies and subjects.

First author	Year	Area	Antiviral drugs	ALT level	Sample size (% male)	Age (years)	ULN of ALT (U/L)	Baseline ALT	HBeAg positive (%)	Baseline HBV DNA	Follow-up time
*Studies comparing the virological outcomes of treated ALT-normal CHB patients with those untreated*											
Perrillo⁣^∗^	2002	Multinational	LAM	Normal	55 (78.1)	Median: 34 (15–73)	NR	Median: 2.2 (0.3–23.4) ULN	100	Median: 98 (LLOD-2264) pg/mL	52 weeks
			IFN		2 (80.9)	Median: 32 (16–70)		Median: 2.4 (0.9–10.1) ULN	100	Median: 111 (LLOD-1322) pg/mL	52 weeks
			LAM + IFN		4 (71.1)	Median: 34 (15–76)		Median: 2.3 (0.8–26.1) ULN	100	Median: 94 (LLOD-786) pg/mL	52 weeks
			Placebo		25 (80.1)	Median: 35 (15–67)		Median: 2.3 (0.4–14.4) ULN	100	Median: 79 (LLOD-1150) pg/mL	52 weeks

Tseng	2014	Taiwan	ETV	Normal	22 (59.1)	Mean: 45 ± 10	NR	Mean: 0.6 ± 0.2 ULN	31.8	Mean: 5.95 ± 1.30 log_10_ copies/mL	52 weeks
			Placebo		20 (55.0)	Mean: 42 ± 12		Mean: 0.6 ± 0.2 ULN	45.0	Mean: 6.31 ± 1.42 log_10_ copies/mL	52 weeks

Lu	2015	China	LDT ⟶ PEG − IFN + ADV	Normal	30 (0)	Mean: 27 ± 3 (total)	40	≤ 1 × ULN	100	> 5 × 10^6^ IU/mL	96 weeks
			LDT ⟶ no treatment		38 (0)			≤ 1 × ULN	100	> 5 × 10^6^ IU/mL	48 weeks

Cao	2017	China	PEG − IFN/+ADV	Normal	94 (66.0)	Mean: 39 ± 10	NR	Mean: 28.3 ± 9.0 U/L	0	< 2000 IU/mL	96 weeks
			No treatment		40 (65.0)	Mean: 40 ± 11		Mean: 25.4 ± 8.4 U/L	0	< 2000 IU/mL	96 weeks

Lim	2019	Singapore	PEG-IFN	Normal	60 (NA)	21–75	NR	≤ 1 × ULN	0	< 2 × 10^4^ IU/mL	48 weeks
			No treatment		30 (NA)	21–75		≤ 1 × ULN	0	< 2 × 10^4^ IU/mL	48 weeks

Zhou	2023	China	ETV/TDF/LDT: continued	Normal	87 (50.6)	Median: 45 (39–50)	50 (male) 40 (female)	Median: 21 (17–25) U/L	0	Median: 3.86 (3.43–4.44) log_10_ IU/mL	60 months
			ETV/TDF/LDT: discontinued		40 (50.0)	Median: 43 (38–54)		Median: 22 (18–28) U/L	0	Median: 4.09 (3.61–4.62) log_10_ IU/mL	57 months
			No treatment		67 (41.8)	Median: 39 (20–49)		Median: 24 (17–30) U/L	0	Median: 3.64 (3.08–4.56) log_10_ IU/mL	48 months

*Studies comparing the antiviral efficacy of CHB patients with normal ALT and those with elevated ALT*											
Perrillo⁣^∗^	2002	Multinational	LAM/IFN/LAM + IFN	Normal	61 (NA)	NR	NR	NR	100	NR	52 weeks
				Elevated	548 (NA)	NR		NR	100	NR	52 weeks

Du	2013	China	LAM/+ADV	Normal	17 (77)	Median: 47 (24–74)	45	Median: 35 (18–45) U/L	35.3	Median: 5.08 (< 0.00–7.70) copies/mL	44.5 months
				Elevated	31 (71)	Median: 44 (26–59)		Median: 86 (46–569) U/L	35.5	Median: 5.77 (3.04–8.89) copies/mL	44.5 months

Chen	2021	China	TDF	Normal + elevated	61 (73.8)	Mean: 44 ± 15	50	Mean: 188.2 ± 97.7 U/L	73.8	Mean: 5.49 ± 1.95 log_10_ IU/mL	24 weeks

Wu	2021	China	ETV	Normal + elevated	127 (61.4)	Mean: 42 ± 11	NR	Mean: 0.7 ± 0.2 ULN	50.4	Mean: 5.3 ± 2.0 log_10_ IU/mL	78 weeks

Xing	2022	China	TAF	Normal	39 (51.3)	Mean: 41 ± 8	50 (male) 40 (female)	Mean: 30.4 ± 11.4 U/L	35.9	Mean: 5.0 ± 2.1 log_10_ IU/mL	24 weeks
				Elevated	40 (60.0)	Mean: 38 ± 9		Mean: 194.3 ± 196.5 U/L	55.0	Mean: 6.7 ± 1.9 log_10_ IU/mL	24 weeks

*Note:* ALT, alanine aminotransferase; ETV, entecavir; IFN, interferon; LAM, lamivudine; LDT, telbivudine; PEG-IFN, pegylated interferon.

Abbreviations: ADV, adefovir dipivoxil; CHB, chronic hepatitis B; HBeAg, hepatitis B envelope antigen; HBV DNA, hepatitis B virus deoxyribonucleic acid; LLOD, lower limit of detectability; NR, not recorded; TAF, terminal aerodrome forecasts; TDF, tenofovir disoproxil fumarate; ULN, upper limit of normal.

⁣^∗^This study included both ALT-normal and ALT-elevated patients and also included both antiviral and nonantiviral patients. Therefore, we analyzed this study as two separate studies.

## Data Availability

The data that supports the findings of this study are available in the Supporting information of this article.

## Supporting Information

Supporting Information 1 Search strategy: Pubmed: ((“Hepatitis B” [MeSH Terms] OR “hepatitis b, chronic” [MeSH Terms] OR “Hepatitis B virus” [MeSH Terms]) AND (“Alanine Transaminase” [MeSH Terms] OR “alanine aminotransferase” [Title/Abstract]) AND (“Treatment Outcome” [MeSH Terms] OR (“Treatment Outcome” [Title/Abstract] OR “Efficacy” [Title/Abstract]))) NOT (“Editorial” [Publication Type] OR “Case Reports” [Publication Type] OR “Review” [Publication Type] OR “Letter” [Publication Type]).

Supporting Information 2 Embase: (‘hepatitis b'/exp OR ‘chronic hepatitis b'/exp OR ‘hepatitis b virus'/exp) AND (‘alanine aminotransferase'/exp OR ‘alanine transaminase':ab,ti) AND (‘treatment outcome' OR ‘treatment outcome':ti,ab, kw OR efficacy:ti,ab,kw) NOT (‘editorial':it OR ‘case reports':it OR ‘review':it OR ‘letter':it).

Supporting Information 3 Web of Science: (((TS = (Hepatitis B)) OR TS = (Chronic Hepatitis B)) OR TS = (Hepatitis B Virus)) AND ((TS = (Alanine Aminotransferase)) OR TS =(Alanine Transaminase)) AND ((TS=(Treatment Outcome)) OR TS = (Efficacy)) NOT (((DT = (Editorial Material)) OR DT = (Review)) OR DT = (Letter)).

Supporting Information 4 Cochrane: ((MeSH descriptor: [Hepatitis B] explode all trees) OR (MeSH descriptor: [Hepatitis B, Chronic] explode all trees) OR (MeSH descriptor: [Hepatitis B virus] explode all trees)) AND ((MeSH descriptor: [Alanine Transaminase] explode all trees) OR ((Alanine Aminotransferase):ti,ab, kw (Word variations have been searched))) AND ((MeSH descriptor: [Treatment Outcome] explode all trees) OR ((Treatment Outcome):ti,ab,kw) OR ((Efficacy):ti,ab,kw)).

Supporting Information 5 Table S1: Preferred Reporting Items for Systematic Reviews and Meta-Analyses (PRISMA) statement.

Supporting Information 6 Table S2: Newcastle–Ottawa Quality Assessment Scale of included studies.

Supporting Information 7 Table S3: Pooled proportions of undetectable HBV DNA before and after trim-and-fill in ALT-normal CHB patients with antiviral therapy.

Supporting Information 8 Table S4: Pooled proportions of HBsAg loss before and after trim-and-fill in ALT-normal CHB patients with antiviral therapy.

Supporting Information 9 Table S5: Pooled proportions of HBeAg loss before and after trim-and-fill in ALT-normal CHB patients with antiviral therapy.

Supporting Information 10 Figure S1: Pooled proportions of HBsAg loss (a) and HBsAg seroconversion (b) in ALT-normal CHB patients with antiviral therapy.

Supporting Information 11 Figure S2: Pooled risk ratios for HBsAg loss (a) and HBsAg seroconversion (b) between the treated group and untreated group.

Supporting Information 12 Figure S3: Subgroup analysis of undetectable HBV DNA stratified by treatment regimen (a) or follow-up time (b) in ALT-normal CHB patients with antiviral therapy.

Supporting Information 13 Figure S4: Subgroup analysis of HBeAg loss stratified by treatment regimen (a) or follow-up time (b) in ALT-normal CHB patients with antiviral therapy.

Supporting Information 14 Figure S5: Subgroup analysis of HBeAg seroconversion stratified by treatment regimen (a) or follow-up time (b) in ALT-normal CHB patients with antiviral therapy.

Supporting Information 15 Figure S6: Subgroup analysis of HBsAg loss stratified by treatment regimen (a) or follow-up time (b) in ALT-normal CHB patients with antiviral therapy.

Supporting Information 16 Figure S7: Subgroup analysis of HBsAg seroconversion stratified by treatment regimen (a) or follow-up time (b) in ALT-normal CHB patients with antiviral therapy.

Supporting Information 17 Figure S8: Funnel plots of HBV DNA before (a) and after (b) trim-and-fill in ALT-normal CHB patients with antiviral therapy.

Supporting Information 18 Figure S9: Funnel plots of HBsAg loss before (a) and after (b) trim-and-fill in ALT-normal CHB patients with antiviral therapy.

Supporting Information 19 Figure S10: Funnel plots of HBeAg loss before (a) and after (b) trim-and-fill in ALT-normal CHB patients with antiviral therapy.

Supporting Information 20 Figure S11: Sensitivity analysis of proportions of undetectable HBV DNA (a), HBeAg loss (b), HBeAg seroconversion (c), HBsAg loss (d), and HBsAg seroconversion (e) in ALT-normal CHB patients with antiviral therapy.

Supporting Information 21 Figure S12: Sensitivity analysis of risk ratios for undetectable HBV DNA (a), HBeAg loss (b), HBeAg seroconversion (c), HBsAg loss (d), and HBsAg seroconversion (e) between the treated group and untreated group.

Supporting Information 22 Figure S13: Sensitivity analysis of risk ratios for undetectable HBV DNA (a), HBeAg loss (b), and HBeAg seroconversion (c) between the ALT-normal and ALT-elevated CHB patients with antiviral therapy.

## References

[1] Hui R. W., Mak L. Y., Fung J., Seto W. K., Yuen M. F. (2024). Prospect of Emerging Treatments for HBV Functional Cure. *Clinical and Molecular Hepatology*.

[2] WHO (2017). Global Hepatitis Report. https://www.who.int/publications/i/item/9789241565455.

[3] Wong R. J., Kaufman H. W., Niles J. K., Kapoor H., Gish R. G. (2023). Simplifying Treatment Criteria in Chronic Hepatitis B: Reducing Barriers to Elimination. *Clinical Infectious Diseases*.

[4] WHO (2015). Guidelines for the Prevention, Care and Treatment of Persons With Chronic Hepatitis B Infection. https://www.who.int/publications/i/item/9789241549059.

[5] WHO (2024). Guidelines for the Prevention, Diagnosis, Care and Treatment for People With Chronic Hepatitis B Infection. https://www.who.int/publications/i/item/9789240090903.

[6] Sinn D. H., Kim S. E., Kim B. K., Kim J. H., Choi M. S. (2019). The Risk of Hepatocellular Carcinoma Among Chronic Hepatitis B Virus-Infected Patients Outside Current Treatment Criteria. *Journal of Viral Hepatitis*.

[7] Terrault N. A., Lok A. S. F., McMahon B. J. (2018). Update on Prevention, Diagnosis, and Treatment of Chronic Hepatitis B: AASLD 2018 Hepatitis B Guidance. *Hepatology*.

[8] Lampertico P., Agarwal K., Berg T. (2017). EASL 2017 Clinical Practice Guidelines on the Management of Hepatitis B Virus Infection. *Journal of Hepatology*.

[9] Sarin S. K., Kumar M., Lau G. K. (2016). Asian-Pacific Clinical Practice Guidelines on the Management of Hepatitis B: A 2015 Update. *Hepatology International*.

[10] You H., Wang F., Li T. (2023). Guidelines for the Prevention and Treatment of Chronic Hepatitis B (Version 2022). *Journal of Clinical and Translational Hepatology*.

[11] Norris J. M., Simpson B. S., Ball R. (2021). A Modified Newcastle-Ottawa Scale for Assessment of Study Quality in Genetic Urological Research. *European Urology*.

[12] Barili F., Parolari A., Kappetein P. A., Freemantle N. (2018). Statistical Primer: Heterogeneity, Random- or Fixed-Effects Model Analyses?. *Interactive Cardiovascular and Thoracic Surgery*.

[13] Perrillo R. P., Lai C. L., Liaw Y. F. (2002). Predictors of HBeAg Loss After Lamivudine Treatment for Chronic Hepatitis B. *Hepatology*.

[14] Du X., Wang J., Shao L. (2013). Histological Improvement of Long-Term Antiviral Therapy in Chronic Hepatitis B Patients With Persistently Normal Alanine Aminotransferase Levels. *Journal of Viral Hepatitis*.

[15] Tseng K. C., Chen C. Y., Tsai H. W. (2014). Efficacy of Entecavir in Chronic Hepatitis B Patients With Persistently Normal Alanine Aminotransferase: Randomized, Double-Blind, Placebo-Controlled Study. *Antiviral Therapy*.

[16] Lu J., Zhang S., Liu Y. (2015). Effect of Peg-Interferon Α-2a Combined With Adefovir In Hbv Postpartum Women With Normal Levels of ALT and High Levels of HBV DNA. *Liver International*.

[17] Cao Z. H., Liu Y. L., Ma L. N. (2017). A Potent Hepatitis B Surface Antigen Response in Subjects With Inactive Hepatitis B Surface Antigen Carrier Treated With Pegylated-Interferon Alpha. *Hepatology*.

[18] Chen P., Wei W., Jin L. (2021). Efficacy and Safety of Tenofovir Alafenamide Fumarate in Nucleoside Analogue Treatment-Naïve Patients With Chronic Hepatitis B. *Experimental and Therapeutic Medicine*.

[19] Wu Z., Ma A. L., Xie Q. (2021). Significant Histological Changes and Satisfying Antiviral Efficacy in Chronic Hepatitis B Virus Infection Patients With Normal Alanine Aminotransferase. Antiviral Therapy Decision in Chronic HBV Patients With Normal ALT. *Clinics and Research in Hepatology and Gastroenterology*.

[20] Zhou J., Wang F. D., Li L. Q., Li Y. J., Wang S. Y., Chen E. Q. (2023). Antiviral Therapy Favors a Lower Risk of Liver Cirrhosis in HBeAg-Negative Chronic Hepatitis B With Normal Alanine Transaminase and HBV DNA Positivity. *Journal of Clinical and Translational Hepatology*.

[21] Lim S. G., Lee G. H., Dan Y. Y. (2019). HBsAg Loss in Inactive Chronic Hepatitis B Carriers is Dependent on Level of qHBsAg and Interferon Response: A Randomised Control Trial. *Hepatology*.

[22] Xing H., Chi X., Sun X., Cheng D., Liu S. (2022). Early Antiviral Efficacy of Tenofovir Alafenamide Fumarate in the Initial Treatment of Chronic Hepatitis B Patients With Normal ALT. *Journal of Hepatology*.

[23] Lin M. H., Li H. Q., Zhu L. (2022). Liver Fibrosis in the Natural Course of Chronic Hepatitis B Viral Infection: A Systematic Review With Meta-Analysis. *Digestive Diseases and Sciences*.

[24] Lee M. H., Yang H. I., Liu J. (2013). Prediction Models of Long-Term Cirrhosis and Hepatocellular Carcinoma Risk in Chronic Hepatitis B Patients: Risk Scores Integrating Host and Virus Profiles. *Hepatology*.

[25] Kim G. A., Lim Y. S., Han S. (2018). High Risk of Hepatocellular Carcinoma and Death in Patients With Immune-Tolerant-Phase Chronic Hepatitis B. *Gut*.

[26] Choi G. H., Kim G. A., Choi J., Han S., Lim Y. S. (2019). High Risk of Clinical Events in Untreated HBeAg-Negative Chronic Hepatitis B Patients With High Viral Load and No Significant ALT Elevation. *Alimentary Pharmacology & Therapeutics*.

[27] Hoang J. K., Yang H. I., Le A. (2016). Lower Liver Cancer Risk With Antiviral Therapy in Chronic Hepatitis B Patients With Normal to Minimally Elevated ALT and No Cirrhosis. *Medicine (Baltimore)*.

[28] Invernizzi F., Viganò M., Grossi G., Lampertico P. (2016). The Prognosis and Management of Inactive HBV Carriers. *Liver International*.

[29] Lee H. W., Kim E. H., Lee J. (2020). Natural History of Untreated HBeAg-Positive Chronic HBV Infection With Persistently Elevated HBV DNA But Normal Alanine Aminotransferase. *Clinical and Translational Gastroenterology*.

[30] Gao W. K., Shu Y. Y., Chen Y. (2022). Effectiveness of Tenofovir Alafenamide in Chronic Hepatitis B Patients With Normal Alanine Aminotransferase and Positive Hepatitis B Virus DNA. *J Clin Transl Hepatol*.

[31] Wei S., Hu M., Chen H. (2022). Effectiveness of Antiviral Treatment in HBeAg-Negative Chronic Hepatitis B Patients With Normal or Mildly Elevated Alanine Aminotransferase: A Retrospective Study. *BMC Gastroenterology*.

[32] Chen X., Zheng X., Wu H., Zhang B., Peng L., Xie C. (2022). Virological Changes of Chronic Hepatitis B Patients With Minimally Elevated Levels of Alanine Aminotransferase: A Meta-Analysis and Systematic Review. *Canadian Journal of Gastroenterology and Hepatology*.

[33] Zhao Q., Liu H., Tang L. (2024). Mechanism of Interferon Alpha Therapy for Chronic Hepatitis B and Potential Approaches to Improve its Therapeutic Efficacy. *Antiviral Research*.

[34] Kim G. A., Lim Y. S., An J. (2014). HBsAg Seroclearance After Nucleoside Analogue Therapy in Patients With Chronic Hepatitis B: Clinical Outcomes and Durability. *Gut*.

[35] Wen C., Wang Y., Tian H. (2023). Clinical Cure Induced by Pegylated Interferon *α*-2b in the Advantaged Population of Chronic Hepatitis B Virus Infection: A Retrospective Cohort Study. *Frontiers in Cellular and Infection Microbiology*.

[36] Liu W. C., Wu I. C., Chiu Y. C. (2020). Genotyping of Immune-Related Loci Associated With Delayed HBeAg Seroconversion in Immune-Active Chronic Hepatitis B Patients. *Antiviral Research*.

[37] Chen H., Fu J. J., Li L., Wang X., Pan X. C. (2024). Risk Factors of Low-Level Viremia in Chronic Hepatitis B Patients Receiving Entecavir Monotherapy: A Retrospective Cohort Study. *Journal of Gastroenterology and Hepatology*.

[38] Gu Y., Zhang Y., Zhang Z. (2024). A Novel Nomogram for Predicting HBeAg Seroclearance in HBeAg-Positive Chronic Hepatitis B Patients Treated With Nucleos(t)ide Analogues. *Annals of Hepatology*.

