# Supplementary material for: Efficacy of Antiviral Therapy in Chronic Hepatitis B Patients With Normal Alanine Aminotransferase: A Systematic Review and Meta-Analysis
Source: Can J Gastroenterol Hepatol. 2025 Mar 8;2025:7689981. doi: 10.1155/cjgh/7689981 (PMC11991825; doi:10.1155/cjgh/7689981)
Supplement: Supporting Information 1 — Search strategy: Pubmed: ((“Hepatitis B” [MeSH Terms] OR “hepatitis b, chronic” [MeSH Terms] OR “Hepatitis B virus” [MeSH Terms]) AND (“Alanine Transaminase” [MeSH Terms] OR “alanine aminotransferase” [Title/Abstract]) AND (“Treatment Outcome” [MeSH Terms] OR (“Treatment Outcome” [Title/Abstract] OR “Efficacy” [Title/Abstract]))) NOT (“Editorial” [Publication Type] OR “Case Reports” [Publication Type] OR “Review” [Publication Type] OR “Letter” [Publication Type]). [file 7689981.f1.docx]

**PubMed Search Strategy:** (("Hepatitis B"[MeSH Terms] OR "hepatitis b, chronic"[MeSH Terms] OR "Hepatitis B virus"[MeSH Terms]) AND ("Alanine Transaminase"[MeSH Terms] OR "alanine aminotransferase"[Title/Abstract]) AND ("Treatment Outcome"[MeSH Terms] OR ("Treatment Outcome"[Title/Abstract] OR "Efficacy"[Title/Abstract]))) NOT ("Editorial"[Publication Type] OR "Case Reports"[Publication Type] OR "Review"[Publication Type] OR "Letter"[Publication Type])
