# Supplementary material for: Efficacy of Antiviral Therapy in Chronic Hepatitis B Patients With Normal Alanine Aminotransferase: A Systematic Review and Meta-Analysis
Source: Can J Gastroenterol Hepatol. 2025 Mar 8;2025:7689981. doi: 10.1155/cjgh/7689981 (PMC11991825; doi:10.1155/cjgh/7689981)
Supplement: Supporting Information 2 — Embase: (‘hepatitis b'/exp OR ‘chronic hepatitis b'/exp OR ‘hepatitis b virus'/exp) AND (‘alanine aminotransferase'/exp OR ‘alanine transaminase':ab,ti) AND (‘treatment outcome' OR ‘treatment outcome':ti,ab, kw OR efficacy:ti,ab,kw) NOT (‘editorial':it OR ‘case reports':it OR ‘review':it OR ‘letter':it). [file 7689981.f2.docx]

**Embase Search Strategy:** ('hepatitis b'/exp OR 'chronic hepatitis b'/exp OR 'hepatitis b virus'/exp) AND ('alanine aminotransferase'/exp OR 'alanine transaminase':ab,ti) AND ('treatment outcome' OR 'treatment outcome':ti,ab,kw OR efficacy:ti,ab,kw) NOT ('editorial':it OR 'case reports':it OR 'review':it OR 'letter':it)
