# Supplementary material for: Efficacy of Antiviral Therapy in Chronic Hepatitis B Patients With Normal Alanine Aminotransferase: A Systematic Review and Meta-Analysis
Source: Can J Gastroenterol Hepatol. 2025 Mar 8;2025:7689981. doi: 10.1155/cjgh/7689981 (PMC11991825; doi:10.1155/cjgh/7689981)
Supplement: Supporting Information 3 — Web of Science: (((TS = (Hepatitis B)) OR TS = (Chronic Hepatitis B)) OR TS = (Hepatitis B Virus)) AND ((TS = (Alanine Aminotransferase)) OR TS =(Alanine Transaminase)) AND ((TS=(Treatment Outcome)) OR TS = (Efficacy)) NOT (((DT = (Editorial Material)) OR DT = (Review)) OR DT = (Letter)). [file 7689981.f3.docx]

**Web of Science Search Strategy:** (((TS=(Hepatitis B)) OR TS=(Chronic Hepatitis B)) OR TS=(Hepatitis B Virus)) AND ((TS=(Alanine Aminotransferase)) OR TS=(Alanine Transaminase)) AND ((TS=(Treatment Outcome)) OR TS=(Efficacy)) NOT (((DT=(Editorial Material)) OR DT=(Review)) OR DT=(Letter))
