# Supplementary material for: Efficacy of Antiviral Therapy in Chronic Hepatitis B Patients With Normal Alanine Aminotransferase: A Systematic Review and Meta-Analysis
Source: Can J Gastroenterol Hepatol. 2025 Mar 8;2025:7689981. doi: 10.1155/cjgh/7689981 (PMC11991825; doi:10.1155/cjgh/7689981)
Supplement: Supporting Information 4 — Cochrane: ((MeSH descriptor: [Hepatitis B] explode all trees) OR (MeSH descriptor: [Hepatitis B, Chronic] explode all trees) OR (MeSH descriptor: [Hepatitis B virus] explode all trees)) AND ((MeSH descriptor: [Alanine Transaminase] explode all trees) OR ((Alanine Aminotransferase):ti,ab, kw (Word variations have been searched))) AND ((MeSH descriptor: [Treatment Outcome] explode all trees) OR ((Treatment Outcome):ti,ab,kw) OR ((Efficacy):ti,ab,kw)). [file 7689981.f4.docx]

**Cochrane Search Strategy:** ((MeSH descriptor: [Hepatitis B] explode all trees) OR (MeSH descriptor: [Hepatitis B, Chronic] explode all trees) OR (MeSH descriptor: [Hepatitis B virus] explode all trees)) AND ((MeSH descriptor: [Alanine Transaminase] explode all trees) OR ((Alanine Aminotransferase):ti,ab,kw (Word variations have been searched))) AND ((MeSH descriptor: [Treatment Outcome] explode all trees) OR ((Treatment Outcome):ti,ab,kw) OR ((Efficacy):ti,ab,kw))
