# Supplementary material for: Efficacy of Antiviral Therapy in Chronic Hepatitis B Patients With Normal Alanine Aminotransferase: A Systematic Review and Meta-Analysis
Source: Can J Gastroenterol Hepatol. 2025 Mar 8;2025:7689981. doi: 10.1155/cjgh/7689981 (PMC11991825; doi:10.1155/cjgh/7689981)
Supplement: Supporting Information 6 — Table S2: Newcastle–Ottawa Quality Assessment Scale of included studies. [file 7689981.f6.docx]

| **Study** | **Selection** | | | | **Comparability** | **Outcome** | | | **Total scores** |
| --- | --- | --- | --- | --- | --- | --- | --- | --- | --- |
|  | **Representativeness of the exposed cohort** | **Selection of the non-exposed cohort** | **Ascertainment of exposure** | **Demonstration that outcome of interest was not present at start of study** |  | **Assessment of outcome** | **Was follow-up long enough for outcomes to occur** | **Adequacy of follow up** |  |
| Perrillo 2002 | 1 | 1 | 1 | 1 | 1 | 1 | 1 | 1 | 8 |
| Tseng 2014 | 1 | 1 | 1 | 1 | 2 | 1 | 1 | 1 | 9 |
| Lu 2015 | 1 | 1 | 1 | 1 | 0 | 1 | 1 | 1 | 7 |
| Cao 2017 | 1 | 1 | 1 | 1 | 2 | 1 | 1 | 1 | 9 |
| Lim 2019 | 1 | 1 | 1 | 1 | 1 | 1 | 1 | 1 | 8 |
| Zhou 2023 | 1 | 1 | 1 | 1 | 1 | 1 | 1 | 0 | 8 |
| Du 2013 | 1 | 1 | 1 | 1 | 2 | 1 | 1 | 1 | 9 |
| Chen 2021 | 1 | 1 | 1 | 1 | 0 | 1 | 0 | 0 | 5 |
| Wu 2021 | 1 | 1 | 1 | 1 | 0 | 1 | 1 | 0 | 6 |
| Xing 2022 | 1 | 1 | 1 | 1 | 1 | 1 | 0 | 1 | 7 |
