# Supplementary material for: Efficacy of Antiviral Therapy in Chronic Hepatitis B Patients With Normal Alanine Aminotransferase: A Systematic Review and Meta-Analysis
Source: Can J Gastroenterol Hepatol. 2025 Mar 8;2025:7689981. doi: 10.1155/cjgh/7689981 (PMC11991825; doi:10.1155/cjgh/7689981)
Supplement: Supporting Information 7 — Table S3: Pooled proportions of undetectable HBV DNA before and after trim-and-fill in ALT-normal CHB patients with antiviral therapy. [file 7689981.f7.docx]

|  | Study number | Undetectable DNA (%) | 95%CI (%) |
| --- | --- | --- | --- |
| Before trim | 8 | 87 | 70 - 98 |
| After trim | 8 + 2 | 94 | 79 - 100 |
