# Supplementary material for: Efficacy of Antiviral Therapy in Chronic Hepatitis B Patients With Normal Alanine Aminotransferase: A Systematic Review and Meta-Analysis
Source: Can J Gastroenterol Hepatol. 2025 Mar 8;2025:7689981. doi: 10.1155/cjgh/7689981 (PMC11991825; doi:10.1155/cjgh/7689981)
Supplement: Supporting Information 8 — Table S4: Pooled proportions of HBsAg loss before and after trim-and-fill in ALT-normal CHB patients with antiviral therapy. [file 7689981.f8.docx]

|  | Study number | HBsAg loss (%) | 95%CI (%) |
| --- | --- | --- | --- |
| Before trim | 5 | 16 | 2 - 38 |
| After trim | 5 + 3 | 39 | 12 - 70 |
