# Supplementary figures and images for: Efficacy of Antiviral Therapy in Chronic Hepatitis B Patients With Normal Alanine Aminotransferase: A Systematic Review and Meta-Analysis
Source: Can J Gastroenterol Hepatol. 2025 Mar 8;2025:7689981. doi: 10.1155/cjgh/7689981 (PMC11991825; doi:10.1155/cjgh/7689981)

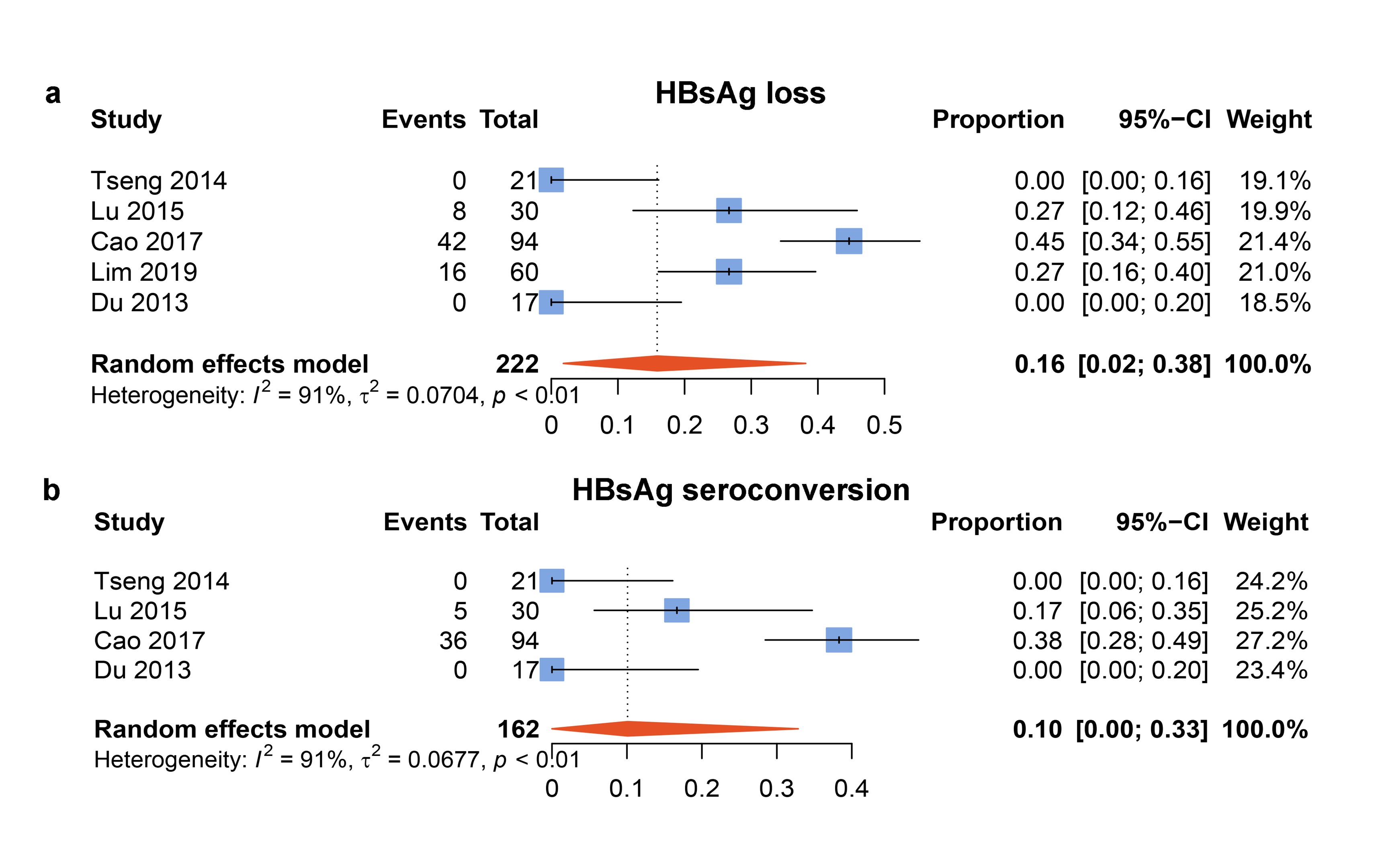

Supplement: Supporting Information 10 — Figure S1: Pooled proportions of HBsAg loss (a) and HBsAg seroconversion (b) in ALT-normal CHB patients with antiviral therapy. [file 7689981.f10.tif]

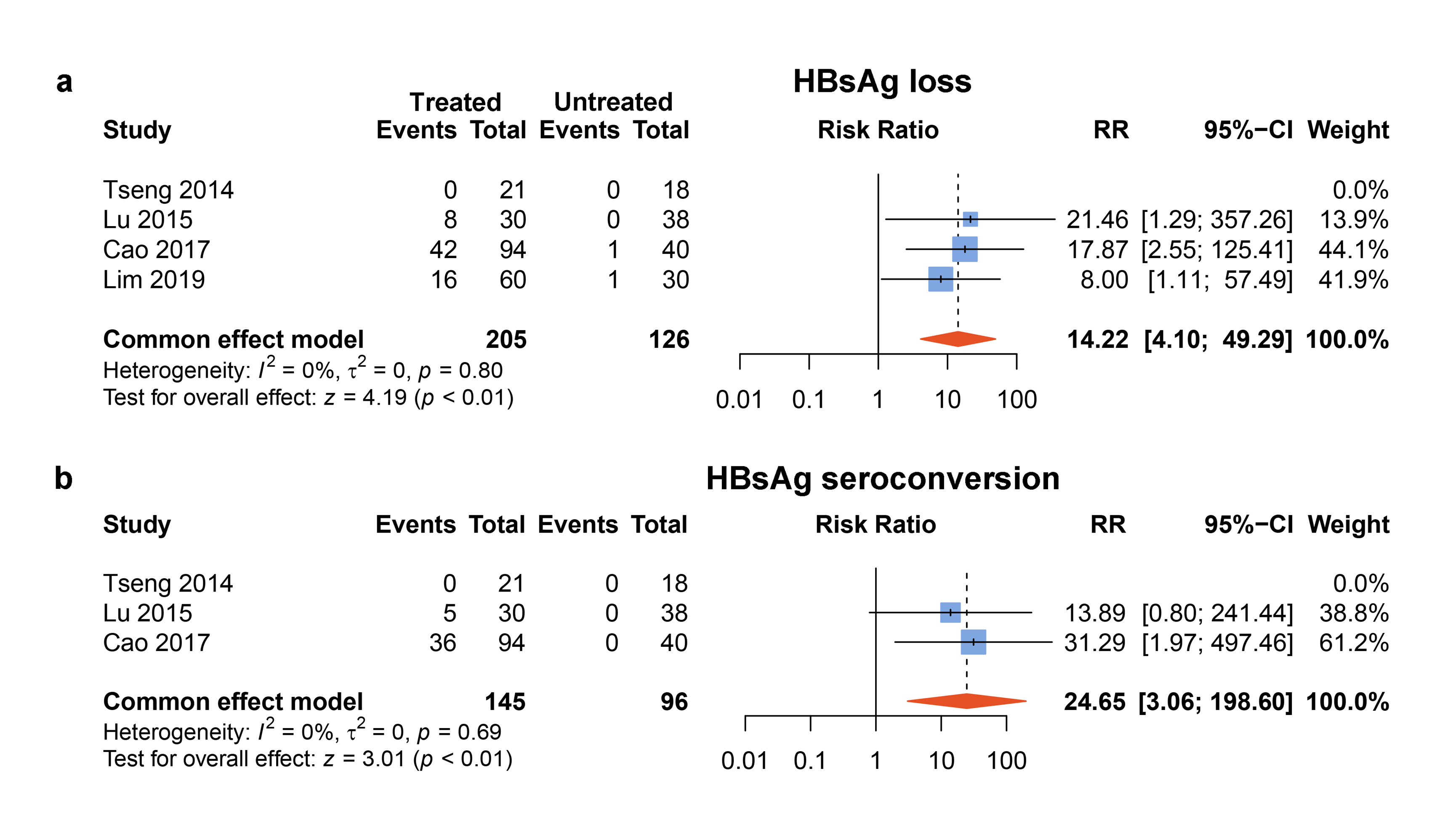

Supplement: Supporting Information 11 — Figure S2: Pooled risk ratios for HBsAg loss (a) and HBsAg seroconversion (b) between the treated group and untreated group. [file 7689981.f11.tif]

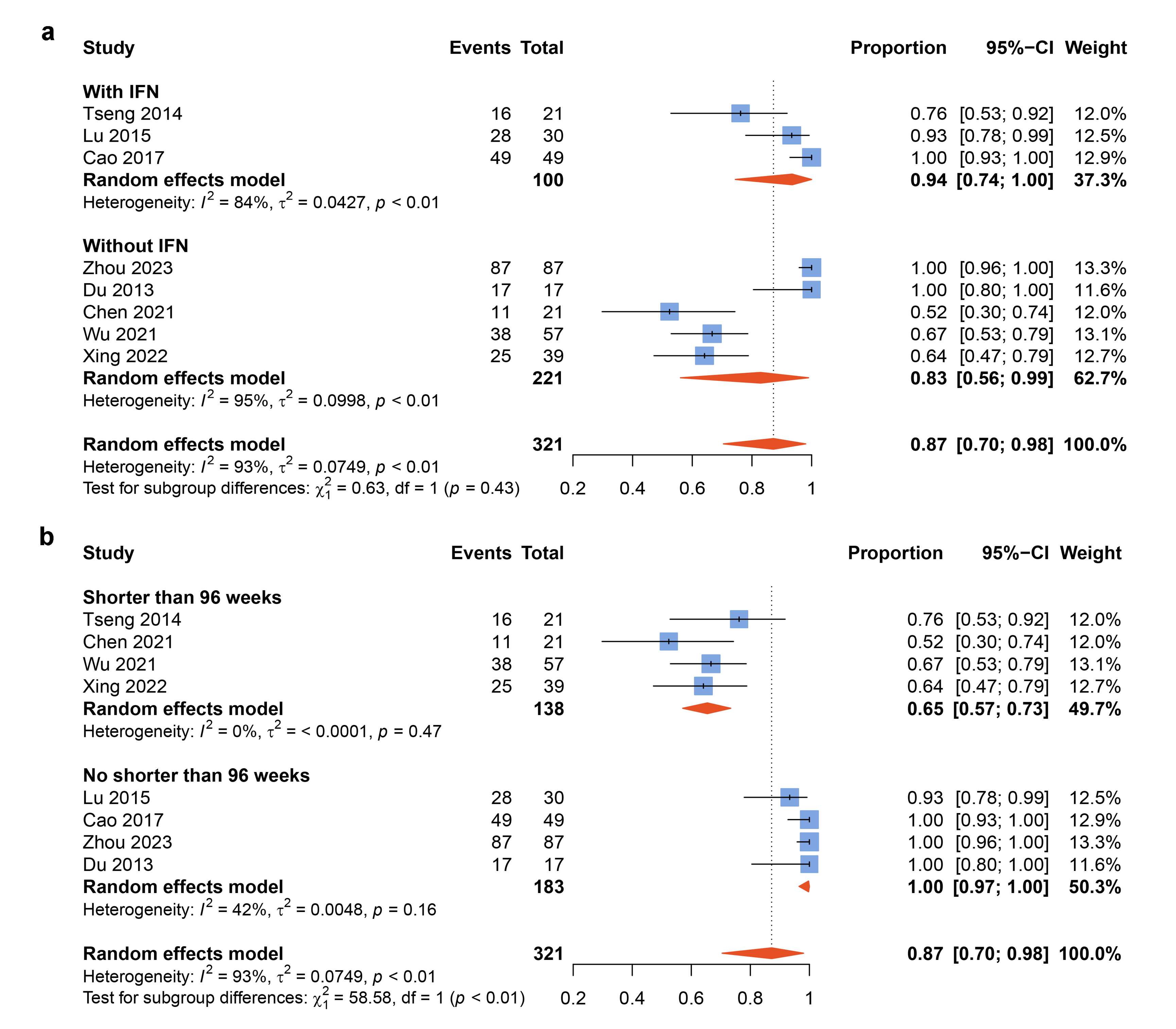

Supplement: Supporting Information 12 — Figure S3: Subgroup analysis of undetectable HBV DNA stratified by treatment regimen (a) or follow-up time (b) in ALT-normal CHB patients with antiviral therapy. [file 7689981.f12.tif]

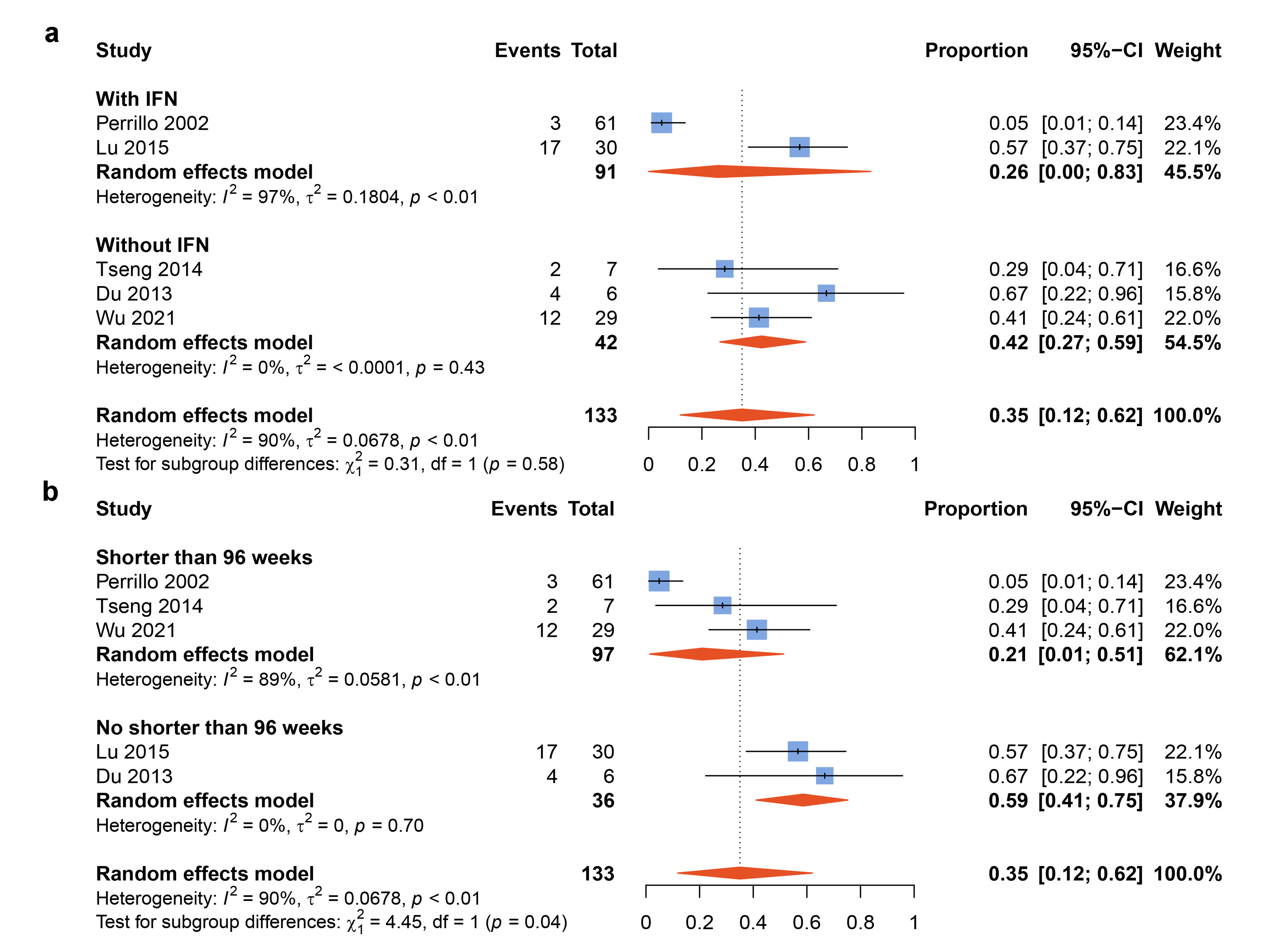

Supplement: Supporting Information 13 — Figure S4: Subgroup analysis of HBeAg loss stratified by treatment regimen (a) or follow-up time (b) in ALT-normal CHB patients with antiviral therapy. [file 7689981.f13.tif]

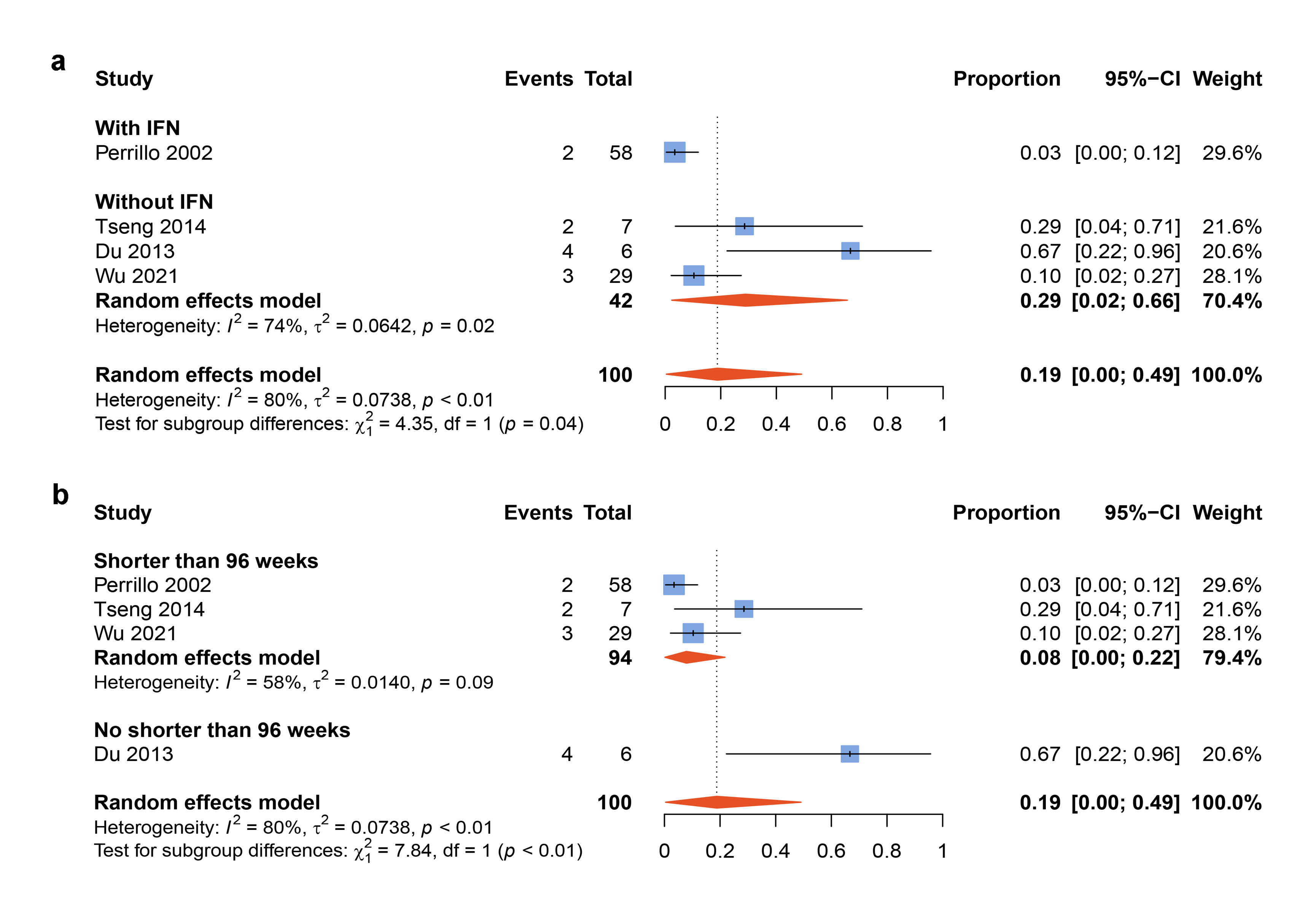

Supplement: Supporting Information 14 — Figure S5: Subgroup analysis of HBeAg seroconversion stratified by treatment regimen (a) or follow-up time (b) in ALT-normal CHB patients with antiviral therapy. [file 7689981.f14.tif]

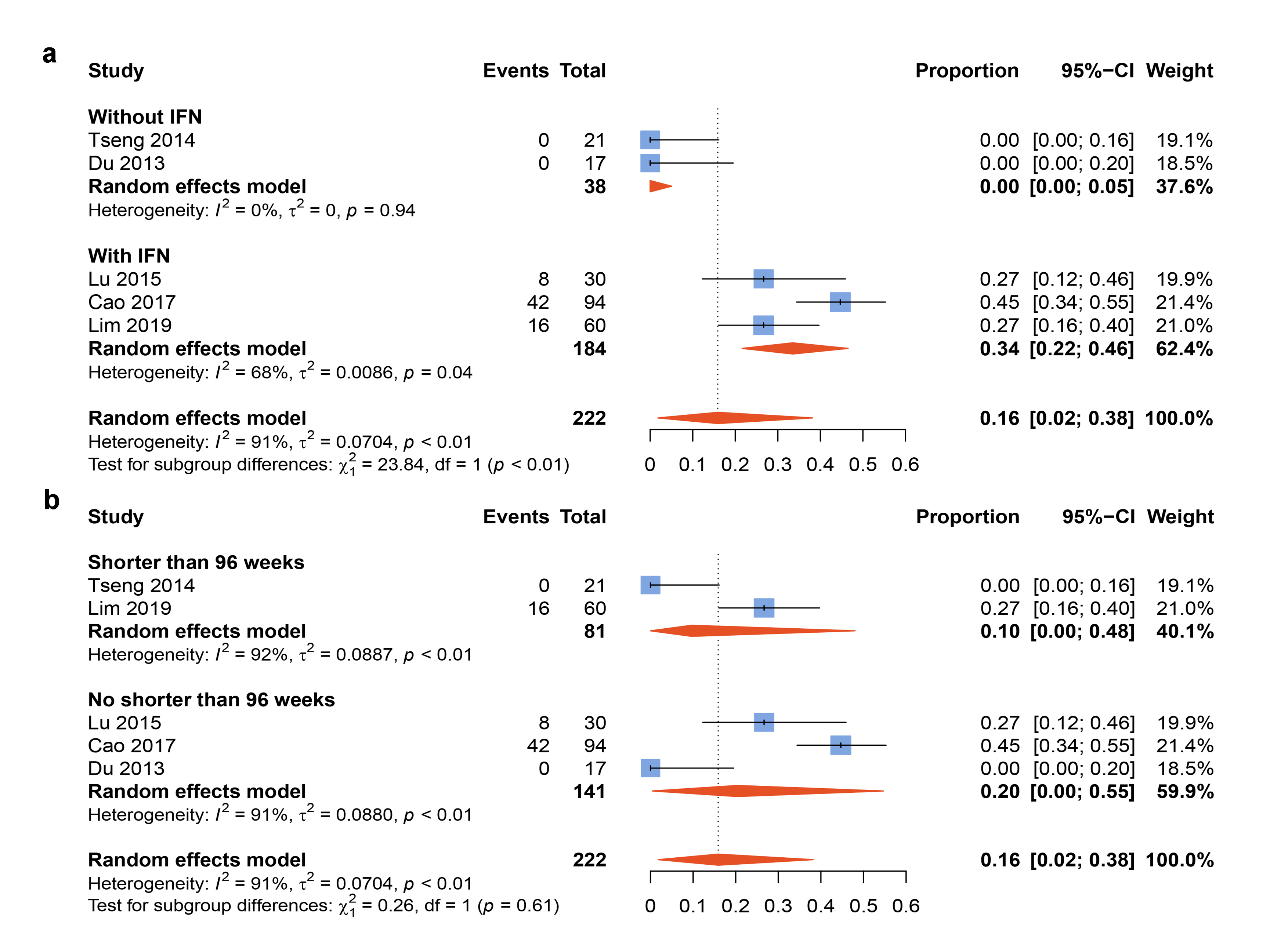

Supplement: Supporting Information 15 — Figure S6: Subgroup analysis of HBsAg loss stratified by treatment regimen (a) or follow-up time (b) in ALT-normal CHB patients with antiviral therapy. [file 7689981.f15.tif]

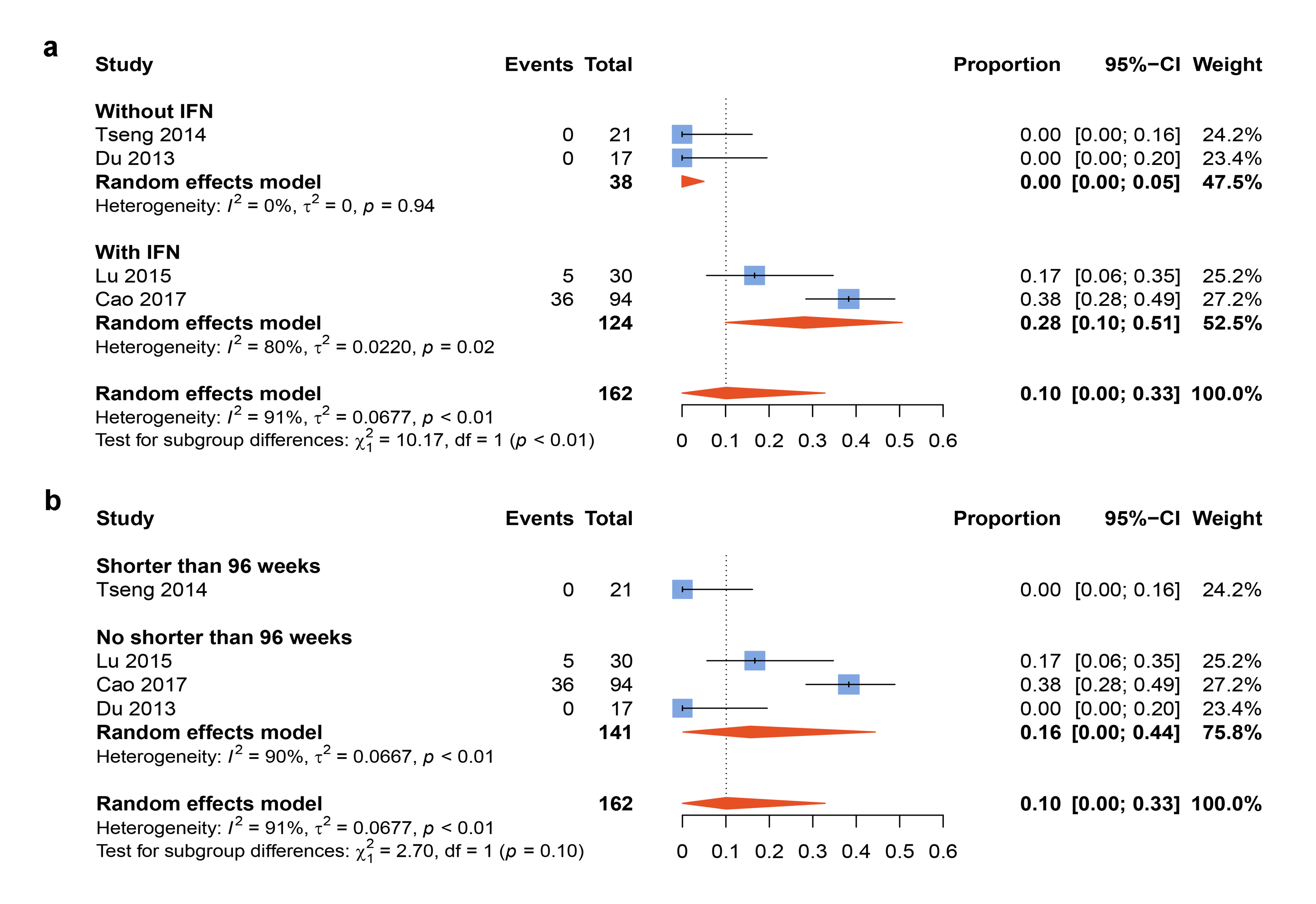

Supplement: Supporting Information 16 — Figure S7: Subgroup analysis of HBsAg seroconversion stratified by treatment regimen (a) or follow-up time (b) in ALT-normal CHB patients with antiviral therapy. [file 7689981.f16.tif]

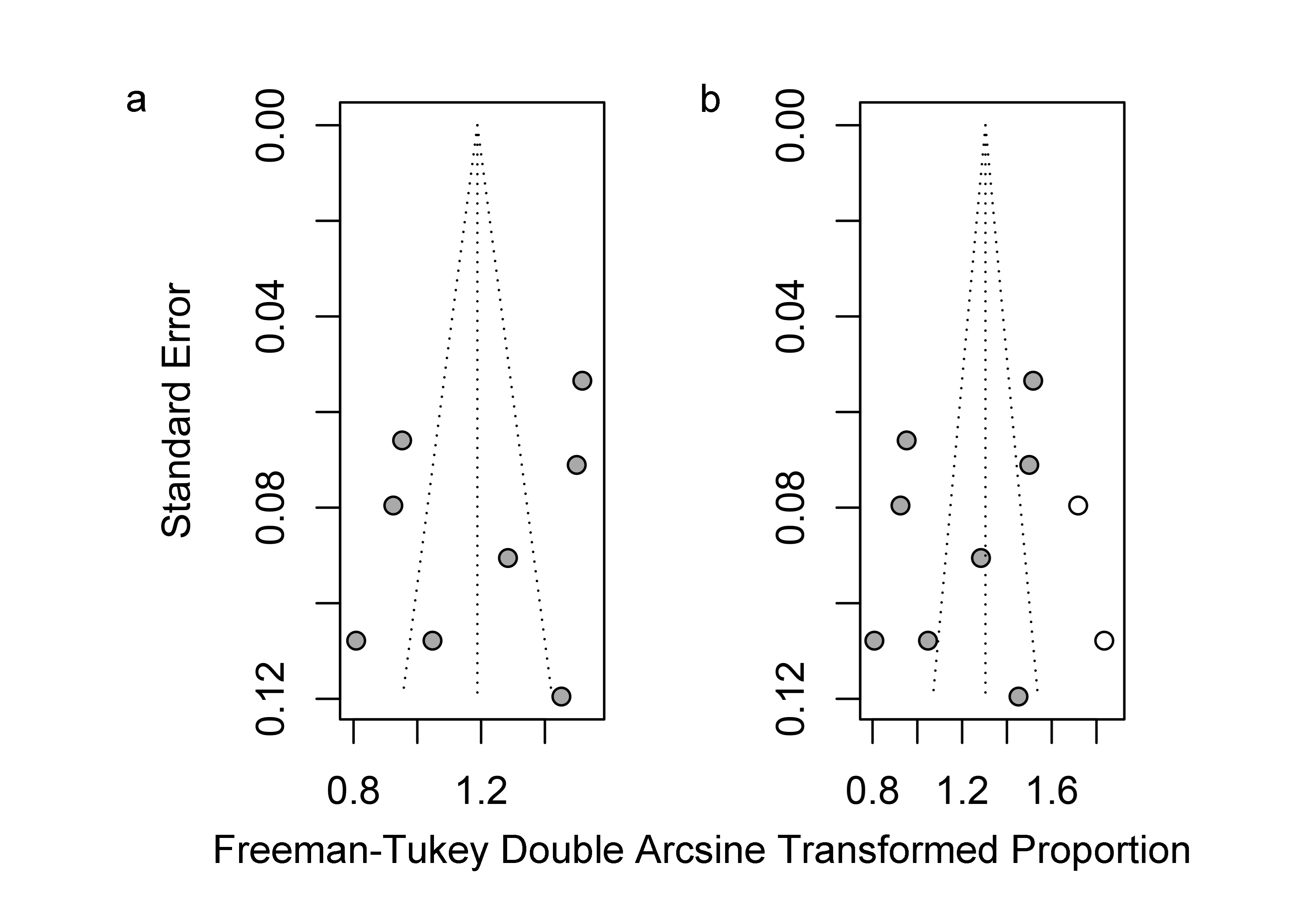

Supplement: Supporting Information 17 — Figure S8: Funnel plots of HBV DNA before (a) and after (b) trim-and-fill in ALT-normal CHB patients with antiviral therapy. [file 7689981.f17.tif]

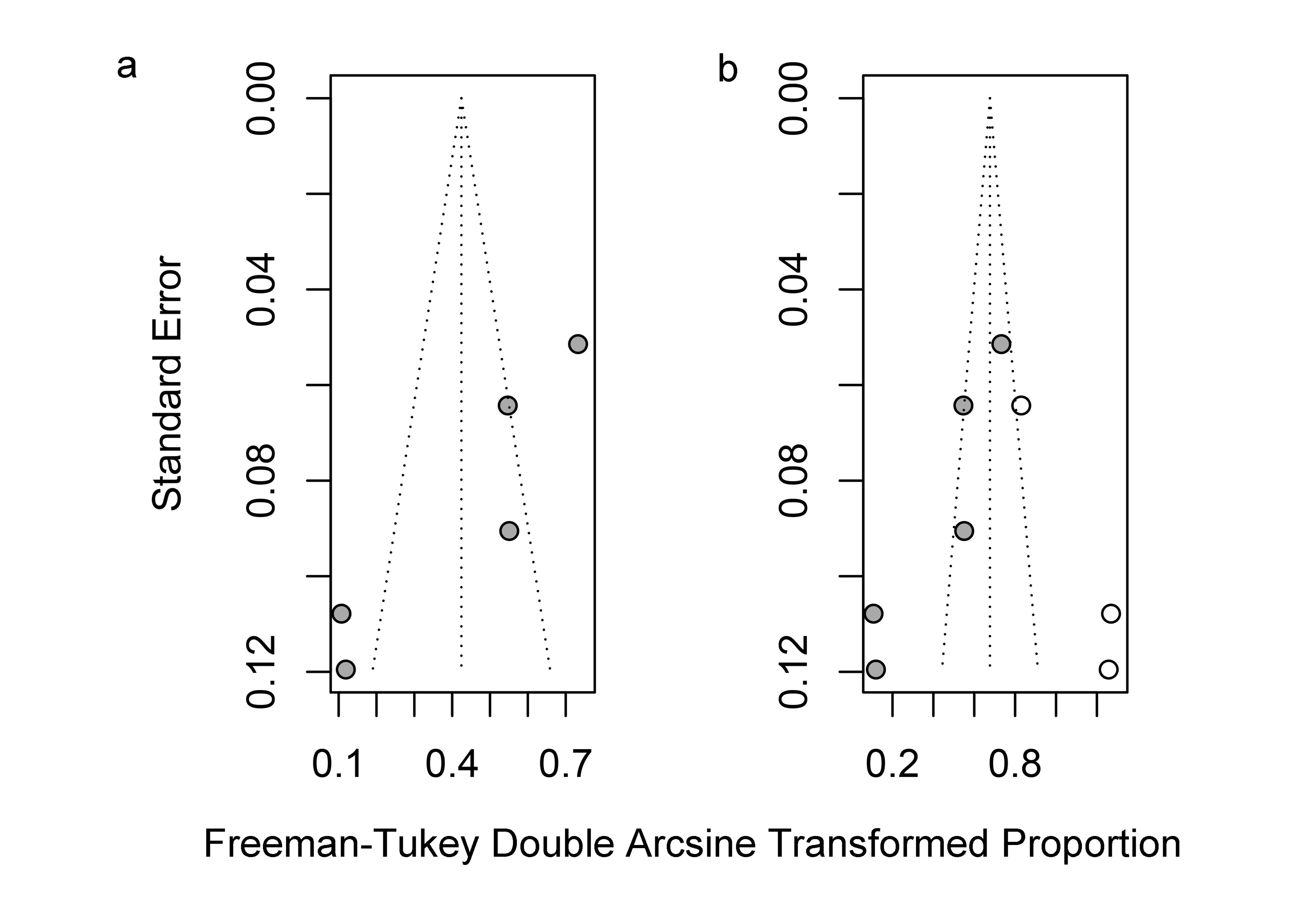

Supplement: Supporting Information 18 — Figure S9: Funnel plots of HBsAg loss before (a) and after (b) trim-and-fill in ALT-normal CHB patients with antiviral therapy. [file 7689981.f18.tif]

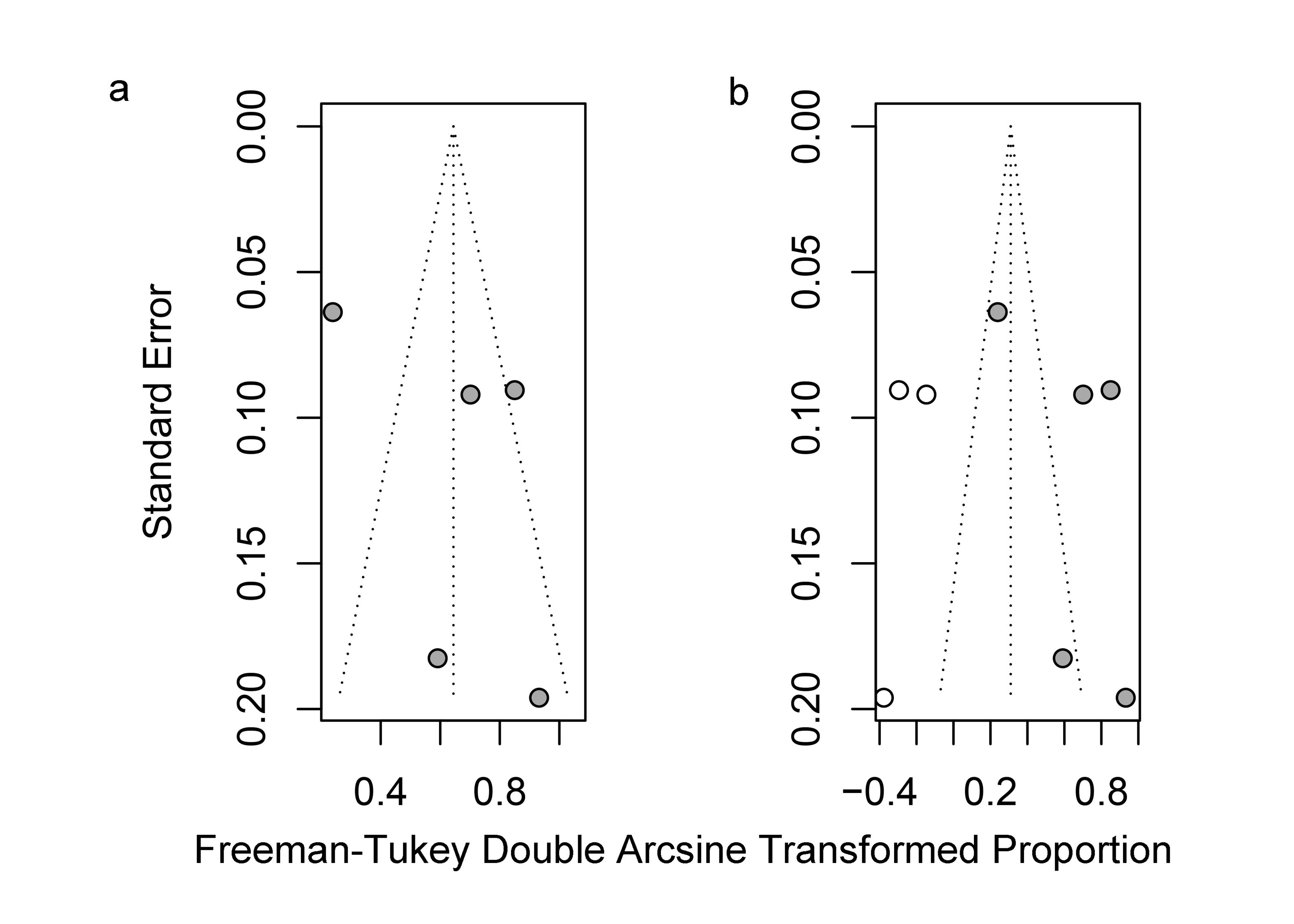

Supplement: Supporting Information 19 — Figure S10: Funnel plots of HBeAg loss before (a) and after (b) trim-and-fill in ALT-normal CHB patients with antiviral therapy. [file 7689981.f19.tif]

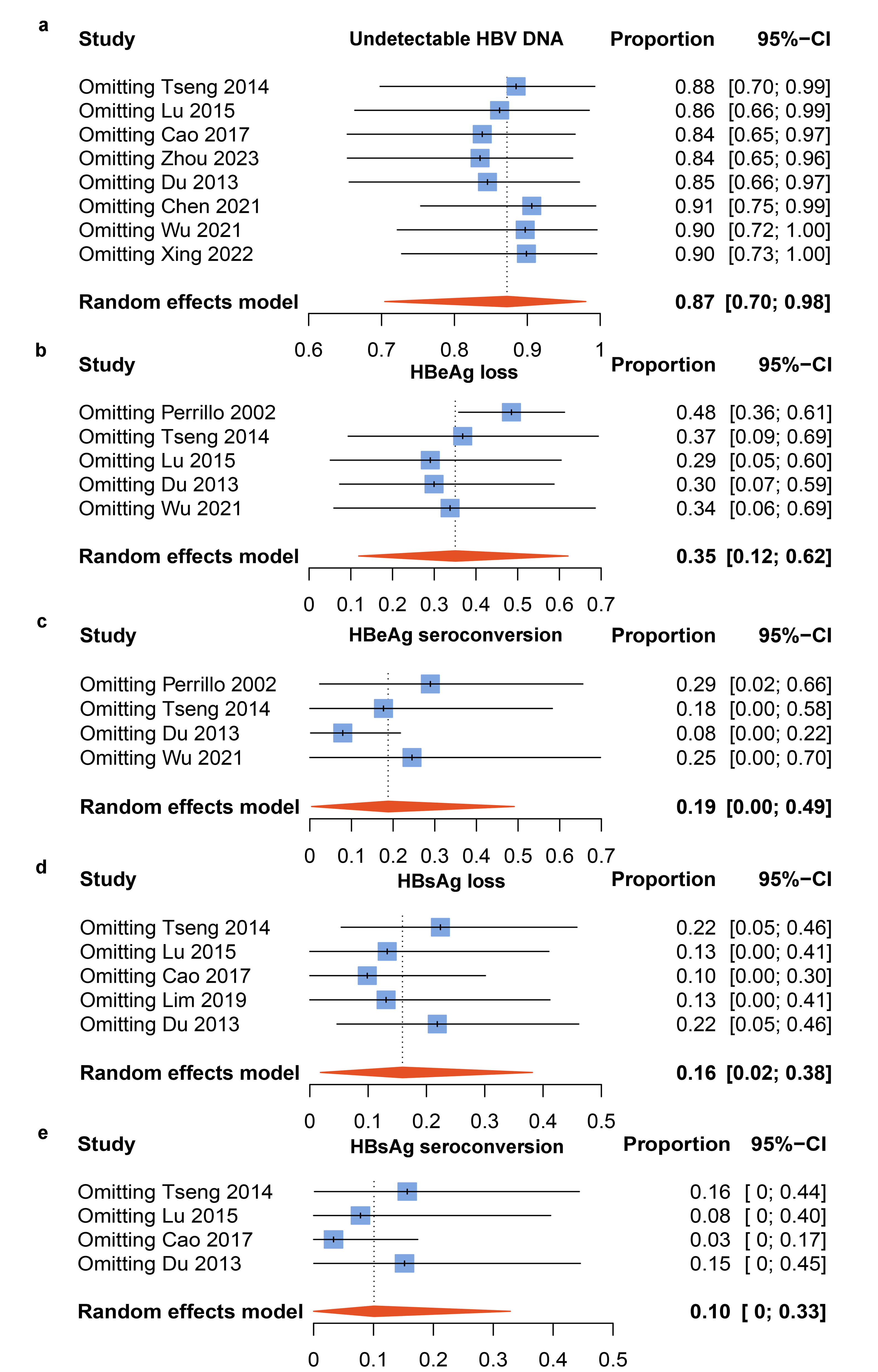

Supplement: Supporting Information 20 — Figure S11: Sensitivity analysis of proportions of undetectable HBV DNA (a), HBeAg loss (b), HBeAg seroconversion (c), HBsAg loss (d), and HBsAg seroconversion (e) in ALT-normal CHB patients with antiviral therapy. [file 7689981.f20.tif]

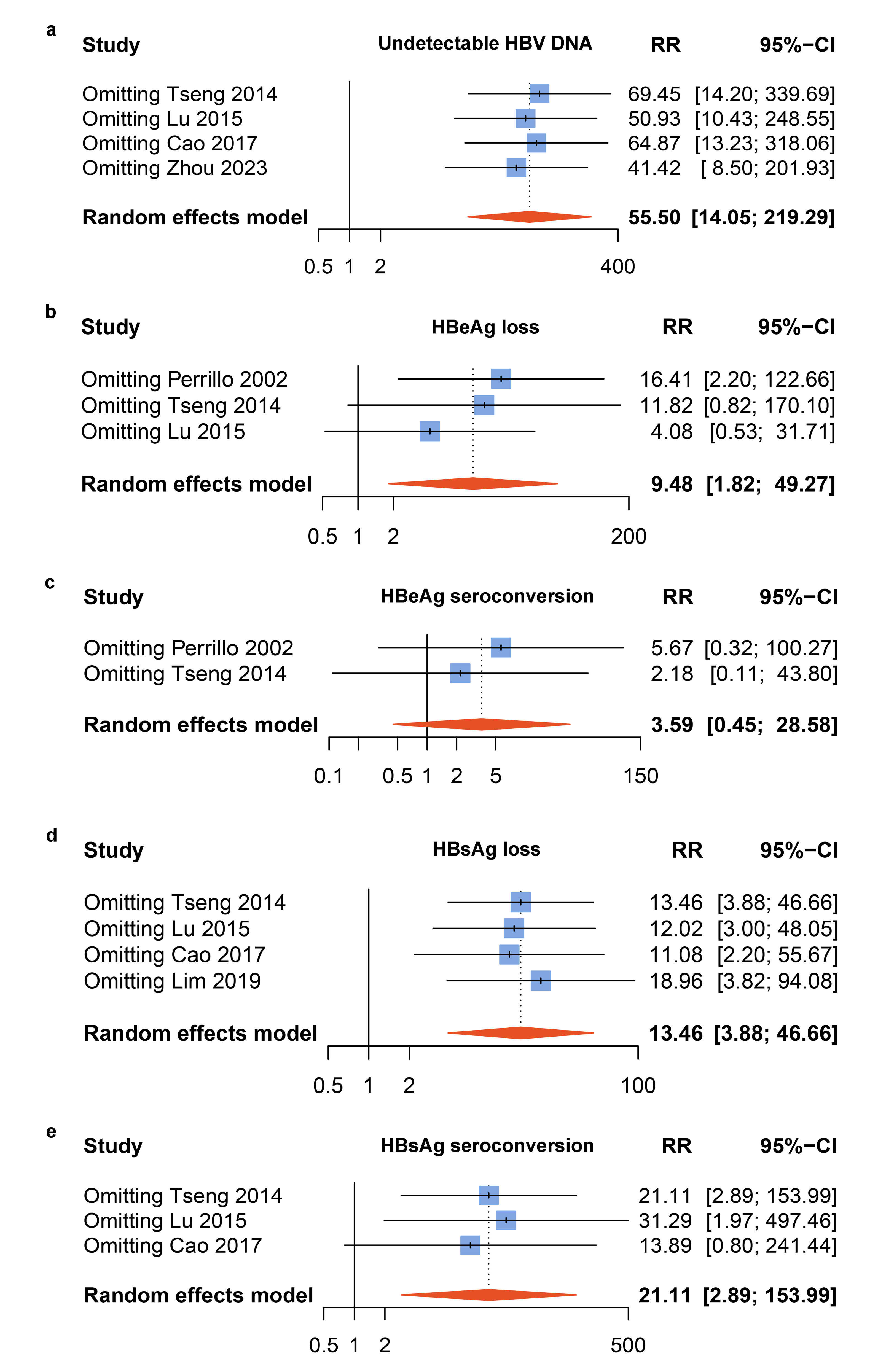

Supplement: Supporting Information 21 — Figure S12: Sensitivity analysis of risk ratios for undetectable HBV DNA (a), HBeAg loss (b), HBeAg seroconversion (c), HBsAg loss (d), and HBsAg seroconversion (e) between the treated group and untreated group. [file 7689981.f21.tif]

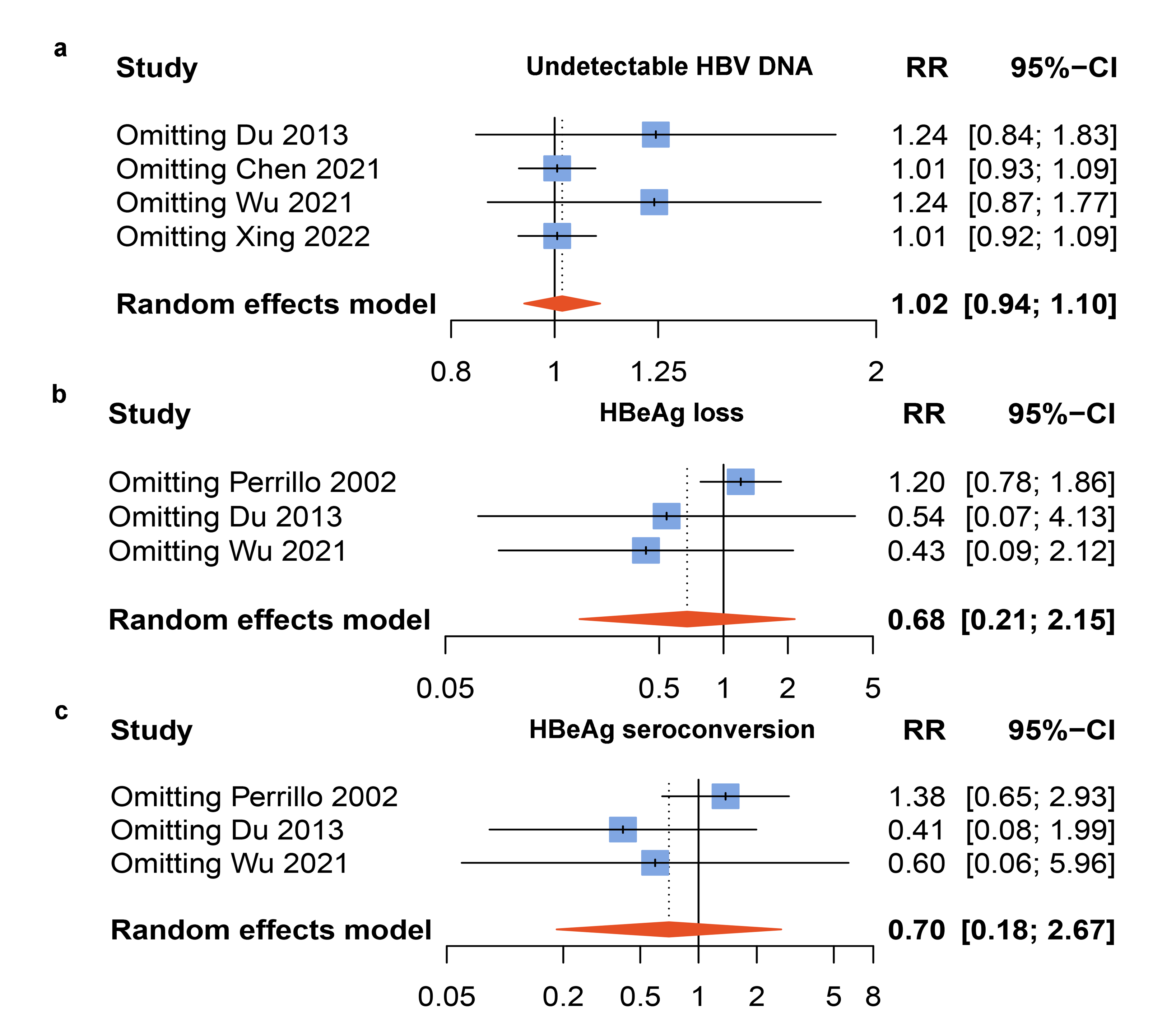

Supplement: Supporting Information 22 — Figure S13: Sensitivity analysis of risk ratios for undetectable HBV DNA (a), HBeAg loss (b), and HBeAg seroconversion (c) between the ALT-normal and ALT-elevated CHB patients with antiviral therapy. [file 7689981.f22.tif]
